# Ensemble learning approach with explainable AI for improved heart disease prediction

**DOI:** 10.3389/fphar.2025.1654681

**Published:** 2025-12-11

**Authors:** Ayomide Adekoya, Faisal Saeed, Wad Ghaban, Sultan Noman Qasem

**Affiliations:** 1 Department of Computer Science, Birmingham City University, Birmingham, United Kingdom; 2 Applied College, University of Tabuk, Tabuk, Saudi Arabia; 3 Computer Science Department, College of Computer and Information Sciences, Imam Mohammad Ibn Saud Islamic University (IMSIU), Riyadh, Saudi Arabia

**Keywords:** cardiovascular disease, explainable AI, heart disease, ensemble learning, XGBoost, ensemble

## Abstract

**Introduction:**

Heart disease remains a leading cause of global morbidity and mortality, motivating the development of predictive models that are both accurate and clinically interpretable. We introduce the Interpretable Ensemble Learning Framework (IELF), which integrates Explainable Boosting Machines (EBM) with XGBoost, SHAP-based explanations, and LIME for enhanced local interpretability.

**Methods:**

IELF was evaluated on two benchmark datasets: Cleveland (n = 303) and Framingham (n = 4,240). Model assessment included 5-fold cross-validation, held-out test sets, calibration, subgroup analyses, and explanation stability evaluation using Kendall’s τ and Overlap@10.

**Results:**

IELF achieved robust discrimination (AUC 0.899, accuracy 88.5% on Cleveland; AUC 0.696, accuracy 82.6% on Framingham) with balanced precision–recall profiles. Compared with EBM, IELF significantly improved recall, F1, and AUC on the Framingham dataset (p < 0.05), while differences versus XGBoost were less consistent. IELF produced transparent feature rankings aligned with established cardiovascular risk factors and stable explanations across folds.

**Discussion:**

IELF is, to our knowledge, the first framework to combine EBM and XGBoost with SHAP and LIME under strict nested cross-validation and calibration procedures. Although headline accuracies are lower than some recent >97% reports, IELF was developed under stricter methodological controls that enhance reproducibility, interpretability, and clinical reliability. These findings position IELF as a trustworthy benchmark for translational AI in cardiovascular risk prediction, complementing high-accuracy but less transparent models.

## 1 Introduction

Cardiovascular disease (CVD) is the leading global cause of mortality, with prevalence projected to rise 46% by 2033 versus 2012 (Virani et al., 2021). Early and accurate prediction is therefore critical for timely intervention and improved patient outcomes (Ghosh et al., 2020; Groenewegen et al., 2020). Traditional statistical approaches have limitations in capturing complex nonlinear interactions, prompting adoption of Machine Learning (ML) methods for diagnosis and risk assessment.

Among ML approaches, ensemble learning shows considerable promise. By leveraging bagging, boosting, and stacking techniques to improve prediction robustness and accuracy over single models (Ali et al., 2019; Mahajan et al., 2023). These methods have been successfully applied to risk stratification and clinical decision support (Naderalvojoud and Hernandez-Boussard, 2024; Atallah and Al-Mousa, 2019). Yet their strength in predictive power often comes at the cost of interpretability. Most ensemble models operate as opaque “black boxes,” concealing the reasoning behind individual predictions and limiting transparency, accountability, and clinician trust. In contrast, clinical decision-making demands interpretability—physicians must be able to understand, verify, and communicate model outputs when guiding patient care (Kernbach and Staartjes, 2020; Aliferis and Simon, 2024).

To address this, explainable AI (XAI) methods have been developed to make complex models more transparent. Techniques such as SHAP provide local and global feature importance, enabling clinicians to identify relevant risk factors (Barredo Arrieta et al., 2020). Recent studies confirm SHAP’s potential in healthcare, offering clinically meaningful explanations that support trust in AI-assisted diagnosis (Albahri et al., 2023). Similarly, Explainable Boosting Machines (EBMs), interpretable models based on generalized additive structures, achieve strong performance while remaining human-readable. They have been applied successfully in critical care contexts such as acute kidney injury prediction (Körner et al., 2024), highlighting their suitability for high-stakes domains.

Despite progress, important gaps remain. Most research focuses either on interpretable models alone or on post-hoc explanations of complex models without rigorous statistical validation. Few efforts combine high-performing ensembles such as XGBoost (Chen and Guestrin, 2016) with inherently interpretable frameworks like EBMs, while simultaneously quantifying the stability of explanations across datasets. Furthermore, calls for clinically viable AI emphasize the need for models that deliver accuracy, interpretability, and reproducibility in tandem (Band, 2023; Albahri et al., 2023; Varga, 2023).

In contrast to prior stacked-ensemble frameworks such as Ghose et al. (2024), which emphasized feature engineering and accuracy optimization, IELF extends this line of work by integrating EBM and XGBoost within a unified interpretable ensemble. Whereas Ghose et al. focused on post-hoc explainability through SHAP, IELF introduces statistical validation of interpretability using quantitative stability metrics (Kendall’s τ, Overlap@10), nested cross-validation, and subgroup fairness and calibration analyses. This establishes IELF as the first statistically validated interpretable ensemble that combines global (SHAP) and local (LIME) explanations to ensure robust, reproducible interpretability rather than accuracy alone. This study addresses these gaps by introducing an Interpretable Ensemble Learning Framework (IELF) for heart disease prediction. The framework integrates EBMs and XGBoost, pairing predictive strength with explainability. Specifically, this work makes three contributions:

1. Hybrid ensemble design: A soft-voting framework combining XGBoost and EBM to capture complementary advantages across multiple heart disease datasets.
2. Quantitative explainability: Integration of SHAP, LIME, and stability metrics (Kendall’s τ, Overlap@10) to validate both global and local feature attribution robustness across folds and subgroups.
3. Rigorous evaluation: Comprehensive performance benchmarking against baseline and state-of-the-art methods, with strict leakage control (nested CV, train-only resampling), uncertainty quantification (95% CIs), calibration, and subgroup fairness analyses.

By aligning predictive performance with transparent explanations, the proposed framework provides an interpretable, clinically relevant decision-support tool for heart disease prediction.

### 1.1 Literature review

Cardiovascular disease (CVD) remains the leading global cause of death, responsible for over 18 million fatalities annually, with incidence projected to rise due to population aging, obesity, and lifestyle-related risk factors (Virani et al., 2021; Roth et al., 2020). Traditional risk models such as the Framingham Risk Score and ASCVD calculator remain widely used for their simplicity and accessibility. However, these tools rely on fixed risk factors and linear assumptions, limiting their ability to capture the non-linear, multifactorial dynamics of modern clinical data (Linardatos et al., 2021; Damen et al., 2019). Recent evidence suggests that these models underperform across diverse populations, raising concerns about generalizability and fairness in real-world practice (Zhang et al., 2023).

Machine Learning (ML) offers an alternative by exploiting large-scale electronic health records, imaging, and biomarkers to enhance prediction accuracy and personalization. ML models have demonstrated superior performance in early diagnosis, mortality prediction, and treatment optimization in cardiovascular medicine (Krittanawong et al., 2020; Javaid et al., 2022; Esteva et al., 2019; Johnson et al., 2023). Deep learning has also achieved success in analyzing medical imaging for coronary artery disease detection (Lia et al., 2025). These advances underscore ML’s potential to surpass conventional risk calculators and support data-driven decision-making. However, ML adoption faces challenges such as limited interpretability, dataset imbalance, and reduced generalizability when validated across heterogeneous populations (Ahmad et al., 2018; Minh et al., 2022; Choi et al., 2023).

### 1.2 Ensemble learning techniques for CVD prediction

Ensemble learning addresses some of these limitations by combining multiple base learners to improve accuracy, robustness, and generalization. By aggregating diverse models, ensembles reduce variance, mitigate bias, and enhance clinical reliability (Dietterich, 2000; Ali et al., 2019). Random Forest, a bagging approach, has shown superior stability and predictive accuracy in CVD classification (Pathan et al., 2022). Boosting algorithms such as XGBoost have achieved strong results in myocardial infarction prediction, outperforming logistic regression in recall and precision (Moore and Bell, 2022). Stacking methods further integrate heterogeneous classifiers through a meta-learner, with recent studies reporting significant gains in heart failure classification (Dalal et al., 2023) and hospital readmission prediction (Ghasemieh et al., 2023). More recently, Tiwari et al. (2022) reported stacked ensembles that improved cardiovascular disease prediction, and Chen et al. (2024) demonstrated ensemble stacking improved discrimination in heart failure patients, while Boadla et al. (2024) showed that integrating clinical and imaging data can improve prognostication of adverse cardiac events.

Despite these strengths, ensemble models such as Random Forest and XGBoost remain limited by poor interpretability, which restricts clinician trust and real-world deployment in cardiovascular workflows.

### 1.3 The challenge of interpretability in ensemble models

While ensemble learning delivers superior predictive performance, its complexity often comes at the cost of interpretability. In clinical practice, where decisions directly affect patient outcomes, this lack of transparency remains a major barrier to adoption. Clinicians require not only accurate outputs but also a clear understanding of the reasoning behind predictions to build trust and support shared decision-making (Kernbach and Staartjes, 2020; Rudin, 2019).

Traditional ensemble methods such as Random Forest and XGBoost are frequently categorized as black-box models, as their aggregated decision pathways are difficult to trace (Lipton, 2018). This opacity undermines utility in high-stakes domains such as cardiology, where regulations demand both accuracy and accountability (Ahmad et al., 2018). Recent evaluations have shown that models with limited transparency face reduced clinical adoption despite superior predictive accuracy (Nguyen et al., 2022).

To address this limitation, post-hoc explainability methods such as SHAP (SHapley Additive exPlanations) and LIME (Local Interpretable Model-Agnostic Explanations) have been introduced. These tools approximate how input variables influence predictions, offering feature attribution scores that make complex models more interpretable (Lundberg and Lee, 2017; Ribeiro et al., 2016). They have been widely used in cardiovascular applications, with studies reporting clinically meaningful insights into factors such as diabetes, cholesterol, and smoking history (Tonekaboni et al., 2019; Karatzia et al., 2022). However, recent work highlights persistent challenges: explanation stability can vary across datasets, robustness is inconsistent in high-dimensional clinical data, and computational overheads remain non-trivial (Farah et al., 2023; Ponce-Bobadilla et al., 2024).

Although SHAP and related methods increase transparency, their variability and lack of statistical validation across diverse patient subgroups limit their reliability for clinical accountability.

### 1.4 Development of interpretable machine learning methods

To address the opacity of black-box models, researchers have advanced intrinsically interpretable approaches. Generalized Additive Models with Pairwise Interactions (GA2Ms) enable direct observation of feature effects, offering greater transparency than conventional ML classifiers (Luo et al., 2019). Building on this foundation, Explainable Boosting Machines (EBMs) extend GA2Ms by using boosted trees for additive modeling, allowing clinicians to analyze predictions without depending solely on post-hoc explanations (Wang et al., 2020). Unlike SHAP or LIME, EBMs integrate interpretability into their structure, which strengthens reliability in clinical settings.

Despite these advantages, EBMs struggle to capture higher-order, high-dimensional interactions, which are often critical in electronic health record (EHR) and imaging datasets (Loh et al., 2022). By contrast, boosting algorithms such as XGBoost model complex nonlinear relationships and optimize accuracy, but their black-box nature hinders clinical acceptance (Rudin, 2019; Chen and Guestrin, 2016).

Recent work has explored hybrid frameworks to reconcile these trade-offs. Al-Alshaikh et al. (2024) showed that integrating EBMs with XGBoost improved accuracy and interpretability in biomarker discovery. Similarly, Chakraborty et al. (2024) combined EBMs, XGBoost, and SHAP to ensure both predictive strength and stable explanations. More recent studies further emphasize the potential of hybrid models: Zhang et al. (2023) demonstrated consistent gains in cardiovascular risk stratification using mixed interpretable-ensemble approaches, while Davoudi et al. (2024) quantified fairness gaps in ensemble ML for heart failure patients, and Li et al. (2023) evaluated and mitigated bias in cardiovascular risk prediction models.

Although EBMs enhance transparency and hybrid methods show promise, existing approaches remain dataset-specific and lack systematic validation of explanation stability across diverse patient cohorts, limiting scalability for clinical practice.

### 1.5 Hybrid approaches combining interpretability and performance

Hybrid frameworks have emerged as promising solutions to balance predictive accuracy and interpretability in cardiovascular disease (CVD) prediction. These approaches combine high-performing ensemble algorithms such as XGBoost with intrinsically interpretable models like EBMs, aiming to produce models that are both accurate and transparent.

XGBoost remains a leading algorithm for structured clinical data due to its ability to capture non-linear interactions, handle missing values, and manage class imbalance effectively (Chen and Guestrin, 2016; Moore and Bell, 2022). However, its black-box nature limits clinician trust. Conversely, EBMs provide feature-level transparency, enabling practitioners to directly observe the influence of risk factors such as hypertension, diabetes, cholesterol, and smoking status on outcomes (Wang et al., 2020; Loh et al., 2022).

Recent research demonstrates the value of combining these paradigms. Al-Alshaikh et al. (2024) reported that an EBM–XGBoost hybrid improved biomarker discovery while preserving interpretability. Chakraborty et al. (2024) extended this by incorporating SHAP-based augmentation to enhance explanation stability across datasets. Similarly, Zhang et al. (2023) showed that hybrid interpretable ensembles achieved better generalizability for cardiovascular risk stratification. Further, Li et al. (2023) highlighted how hybrid interpretable boosting improved prediction consistency in imbalanced CVD cohorts. Ghose et al. (2024) recently proposed a hybrid explainable ensemble integrating EBM, XGBoost, and SHAP with stacked meta-learning, achieving strong accuracy but limited analysis of explanation stability or reproducibility. Their approach inspired aspects of the current work, which extends hybrid interpretability through quantitative stability validation and detailed runtime reproducibility documentation.

Empirical evidence reinforces this trend. Atallah and Al-Mousa (2019) achieved 90% accuracy on the Cleveland Heart Disease dataset using a voting-based ensemble. Dalal et al. (2023) reported that stacked ensembles outperformed standalone classifiers in heart failure prediction, while Ghasemieh et al. (2023) showed strong recall using XGBoost-based stacking for hospital readmission. These findings suggest that hybrid and stacked methods hold significant potential for clinical adoption.

Despite strong results, current hybrid ensembles remain constrained by dataset-specific designs, lack rigorous subgroup validation, and face challenges with scalability and workflow integration into real-world clinical environments.

Closest to our focus, Mienye and Jere (2024) integrated Random Forest, AdaBoost, and XGBoost with Bayesian optimization for hyperparameter tuning and utilized SHAP for post-hoc interpretability and reached extremely high sensitivity and specificity on the Cleveland (0.971/0.989) and Framingham (0.921/0.975) datasets. Though their work points to the promise of optimized ensembles with SHAP interpretations, their focus is maintaining maximal predictive performance. In comparison, the current work introduces IELF, a combination of EBM and XGBoost, that places intrinsic interpretability, explanation stability, calibration, and subgroup fairness at its forefront, substantiated under nested cross-validation with strict leakages control. In doing so, IELF fills the gap of accurate ensemble methods that prioritize reproducibility and clinical trustworthiness alongside discriminative power.

### 1.6 Identified research gap

The literature validates, machine learning, specifically ensemble techniques like Random Forest, XGBoost, and stacked classifiers, is successfully outperforming older cardiovascular risk models. Meanwhile, interpretable techniques like EBMs and GA2Ms boost transparency, while post-hoc techniques like SHAP build clinical trust by explaining feature contribution.

Nonetheless, there is inevitably a lingering tradeoff between accuracy and interpretability. State-of-the-art ensembles such as XGBoost obtain higher predictive results but are black boxes that enforce limited clinician trust and utilization. In exchange, interpretable models such as EBMs disclose decision pathways but systematically underperform when confronted with complicated feature interactions in the electronic health records and multi-cohort sets.

More recent work by Mienye and Jere (2024) points to this trade-off: their ensembles optimized with SHAP achieved remarkable prediction performance on the Cleveland and Framingham data sets, yet their strategy desired to optimize the performance at the cost of forgoing reporting calibration, subgroup fairness, and explanation stability. It underlines the main drawback of the research at hand, that models that excel under carefully controlled conditions often forego the required reproduction and reliability tests that are needed prior to clinical application. Hybrid approaches have begun to emerge, but they are often dataset-specific, need to be described qualitatively, or forgo strict statistical verification of explanation strength for subgroups of patients.

Additional challenges compound this gap:

- The data imbalance persists to prevent accurate prediction of the minority outcomes.
- Generalizability is rarely tested on various populations, raising questions of equity.
- Clinical workflow integration is typically overlooked, therefore inhibiting real-world deployment.
- Leakage control is not enforced equally, and pre-split resampling and feature engineering threaten over-performance.
- Subgroup fairness and calibration are underreported; hence real-world reliability is unpredictable.

Collectively, these constraints spotlight the requirement for such a framework as the IELF that not only achieves competitive prediction capability but also incorporates basic elements of intrinsic interpretability, explanation stability, calibration, and fairness into leakage-safe validation protocols, hence toward reliable and clinically valid artificial intelligence for the prediction of cardiovascular disease.

## 2 Materials and methods

### 2.1 Datasets

This study employed two complementary cardiovascular disease (CVD) datasets to ensure both benchmark comparability and real-world clinical representativeness.

### 2.2 Framingham Heart Study dataset

The Framingham Heart Study is a longitudinal cohort established in 1948 to identify constitutional and environmental risk factors for CVD. For this work, a subset comprising 4,240 participants was used, including 15 predictors spanning demographic, clinical, and behavioural domains such as age, sex, cholesterol, blood pressure, diabetes status, smoking, and medication history. Challenges included a high rate of missingness in metabolic variables (e.g., glucose, cholesterol, heart rate) and heterogeneity in medication use. Furthermore, the dataset is class imbalanced, with far fewer positive CVD outcomes than negatives, necessitating resampling strategies to avoid biased models.

### 2.3 Cleveland Heart disease dataset

The Cleveland dataset, sourced from the UCI Machine Learning Repository, contains 303 patient records with 13 clinical attributes, including chest pain type, resting blood pressure, cholesterol, fasting blood sugar, resting electrocardiographic results, and maximum heart rate achieved. The dataset labels individuals with varying degrees of CVD severity, though this was binarized into presence/absence of disease for the predictive task. Unlike Framingham, the Cleveland dataset is relatively balanced across outcome classes, making it a widely used benchmark for algorithmic comparison.

### 2.4 Dataset harmonization

To ensure comparability, preprocessing steps were aligned across the two datasets. Variable schema differences were standardized (e.g., binary encoding of smoking and diabetes status, categorical encoding of chest pain type and medication use). Where features existed in one dataset but not the other, alignment was achieved through harmonization of overlapping variables and documentation of divergences. This approach ensured that both datasets could contribute to a consistent pipeline for training and evaluation.

As summarized in Table 1, Cleveland is nearly complete except for angiographic variables (*ca*, *thal*), while Framingham exhibits more substantial missingness in variables such as glucose and cholesterol. Although both datasets are widely used in CVD prediction research, limitations remain. The Framingham dataset is outdated in demographic diversity, while Cleveland’s small size restricts generalizability. Importantly, cross-dataset harmonization cannot fully overcome structural differences, meaning that interpretability results may vary by cohort and require external clinical validation.

**TABLE 1:** Dataset summary and missingness. Cohort size, dimensionality, and variable-level missingness prior to preprocessing. The Framingham dataset had substantial missingness in metabolic variables (e.g., glucose, cholesterol), whereas Cleveland was nearly complete except for angiographic variables (ca, thal).

Dataset	N (rows)	p (predictors)	Example variables	Missing (selected)
Cleveland (UCI)	303	13	age, sex, cp, trestbps, chol, fbs, restecg, thalach, exang, oldpeak, slope, ca, thal	ca: 4; thal: 2; all others: 0
Framingham	4,240	15	sex, age, education, currentSmoker, cigsPerDay, BPMeds, prevalentStroke, prevalentHyp, diabetesetc.	glucose: 388; education: 105; totChol: 50; BPMeds: 53; cigsPerDay: 29; BMI: 19; HR: 1

Taken together, Tables 1, 2 highlight the complementary nature of the two cohorts: Cleveland provides a relatively small but balanced benchmark dataset, while Framingham offers a larger, more heterogeneous, and highly imbalanced cohort. These structural differences necessitated a carefully aligned preprocessing pipeline to ensure comparability and fairness across models.

**TABLE 2:** Class Balance and Data Splits. Outcome distribution and data partitioning. Framingham exhibits substantial imbalance; SMOTE was applied only to Framingham during training (see Figure 1).

Dataset	Negative (chd = 0)	Positive (chd = 1)	Imbalance ratio	Train shape (X)	Test shape (X)	Processed train shape	Processed test shape
Cleveland	164	139	1.18:1 (balanced)	(242, 13)	(61, 13)	(242, 25)	(61, 25)
Framingham	3,596	644	5.58:1 (imbal.)	(3,392, 15)	(848, 15)	(3,392, 20)	(848, 20)

### 2.5 Data preprocessing

The preprocessing workflow (Figure 2) was designed to address missing values, class imbalance, and schema differences between datasets while preparing features for downstream modeling and explainability. Key steps included:

- Missing Data Handling: Continuous variables (e.g., glucose, cholesterol) with moderate missingness in Framingham were imputed using median values, while binary/categorical fields (e.g., BPMeds, education) were imputed with mode. Cleveland required minimal imputation (only ca and thal).
- Feature Harmonization: Variable names and encodings were standardized (e.g., binary indicators for smoking and diabetes, categorical encoding for chest pain and medication use).
- Resampling: To address Framingham’s 5.58:1 class imbalance, SMOTE oversampling was applied only on the training set. Cleveland was left unchanged due to its natural balance.
- Scaling: Continuous variables were standardized (z-score normalization) to avoid bias toward high-magnitude predictors.
- Partitioning: Both datasets were split into train (80%) and test (20%) subsets with stratification to preserve class distributions.

**FIGURE 1:**
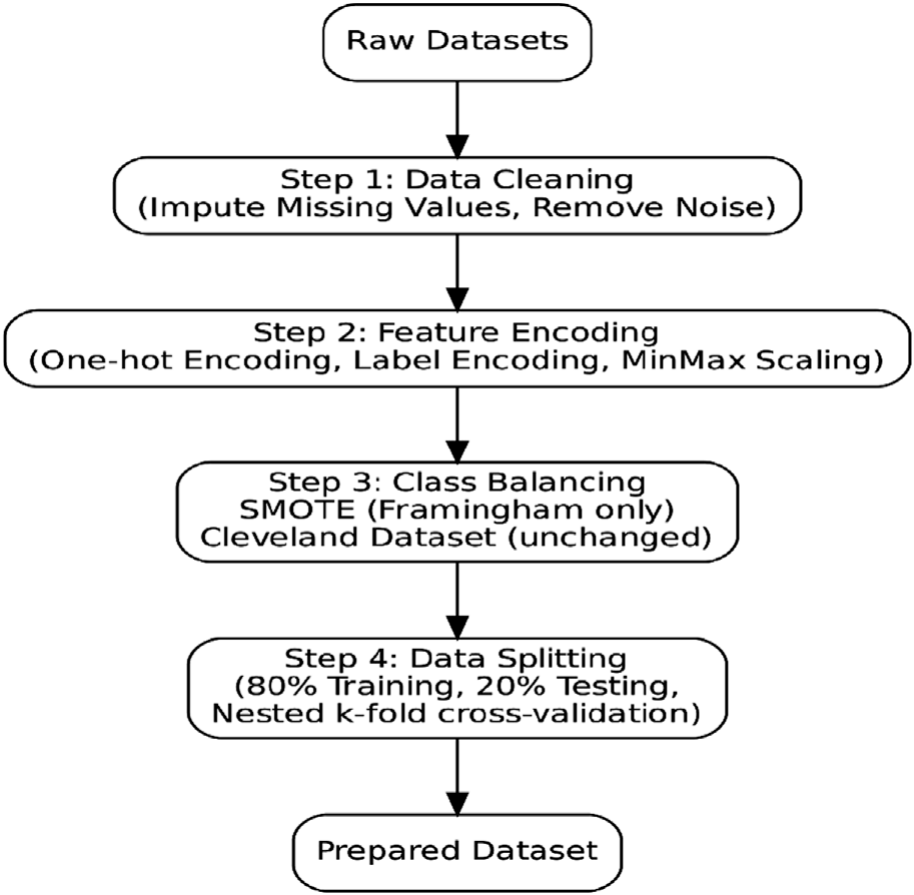
Data preprocessing workflow applied to the Framingham and Cleveland Datasets, including handling of missing values, encoding/scaling, class balancing, and partitioning for training/testing.

Figure 1 illustrates the preprocessing pipeline, showing sequential stages from raw data ingestion through imputation, encoding, class balancing, and partitioning.

**FIGURE 2:**
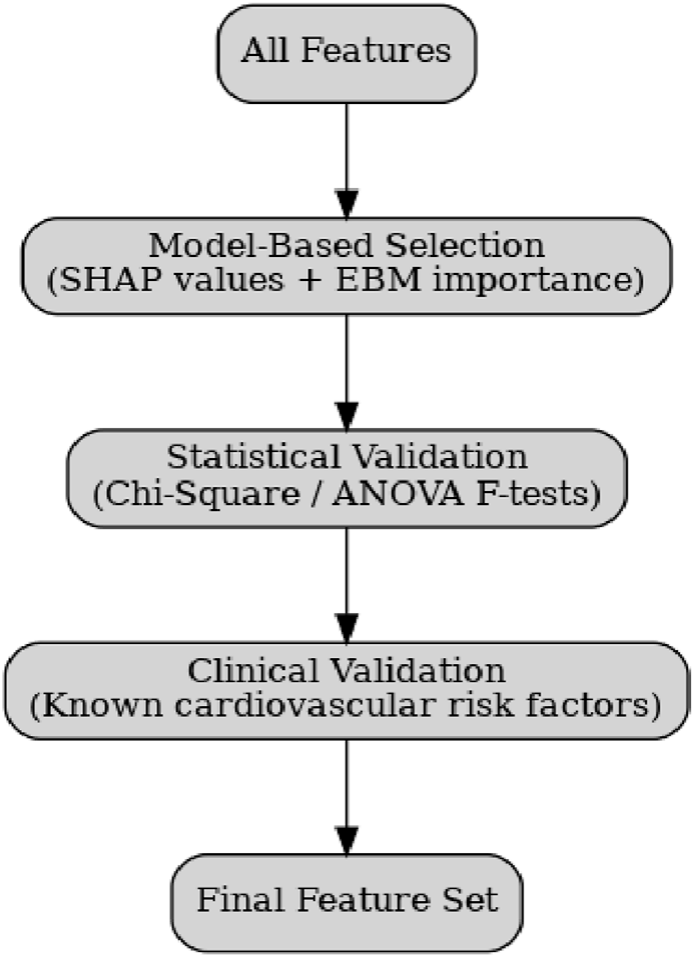
Hybrid feature selection workflow integrating model-based rankings (SHAP, EBM), statistical tests, and clinical validation to produce the final set of features.

While preprocessing mitigates missingness, scaling disparities, and imbalance, these methods cannot fully resolve dataset-inherent biases. SMOTE may introduce synthetic patterns that diverge from true clinical distributions, while harmonization risks obscuring population-specific nuances.

### 2.6 Feature selection

Effective feature selection was critical to improving predictive accuracy, reducing computational complexity, and ensuring that retained features align with established cardiovascular risk factors. The study employed a hybrid strategy combining model-driven, statistical, and clinical validation approaches.

#### 2.6.1 Model-based selection

- SHAP (Shapley Additive Explanations) values were extracted from the XGBoost model to quantify each feature’s marginal contribution to predictions. This allowed ranking of features by global importance, while also supporting local interpretability for patient-level decisions.
- EBM Feature Importance scores were derived from additive function terms, capturing both univariate and pairwise interactions. Features with consistently high EBM contributions were prioritized for inclusion.

#### 2.6.2 Statistical validation

- Chi-Square Tests (for categorical variables) and ANOVA F-tests (for continuous variables) were applied to evaluate the statistical association between individual features and the CVD outcome.
- Features failing to achieve statistical significance across both datasets were discarded to avoid noise and redundancy.

#### 2.6.3 Clinical relevance filtering

To prevent overfitting to dataset-specific artifacts, the feature subset was cross-checked against established CVD risk markers, such as age, cholesterol, systolic blood pressure, diabetes status, and smoking. Features with low predictive or clinical validity were excluded, ensuring interpretability and domain alignment.

**FIGURE 3:**
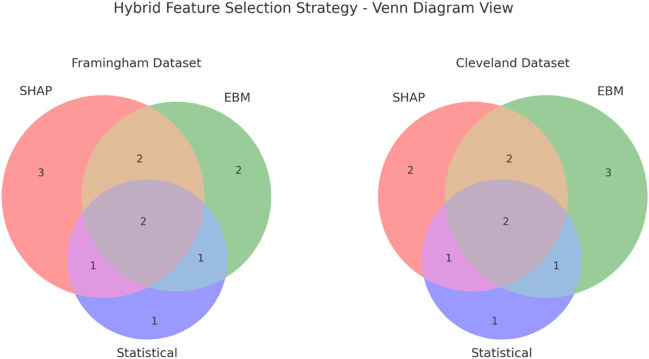
Venn diagram representation of the hybrid feature selection strategy for the Framingham (left) and Cleveland (right) datasets. Each circle represents features retained by a single method, SHAP, EBM, or Statistical tests. Overlaps indicate features cons consistently identified across multiple methods. The central intersection shows the final consensus features retained after clinical validation.

Figure 3 depicts the feature selection framework, combining SHAP rankings, EBM contributions, statistical tests, and clinical validation into a unified process for identifying the final feature set.

### 2.7 Final feature set

The resulting features represented a balanced overlap of those identified by SHAP, EBM, and statistical tests, with additional reinforcement from clinical literature. This multi-layered selection process enhanced robustness and credibility across both the Framingham and Cleveland datasets.

**FIGURE 4:**
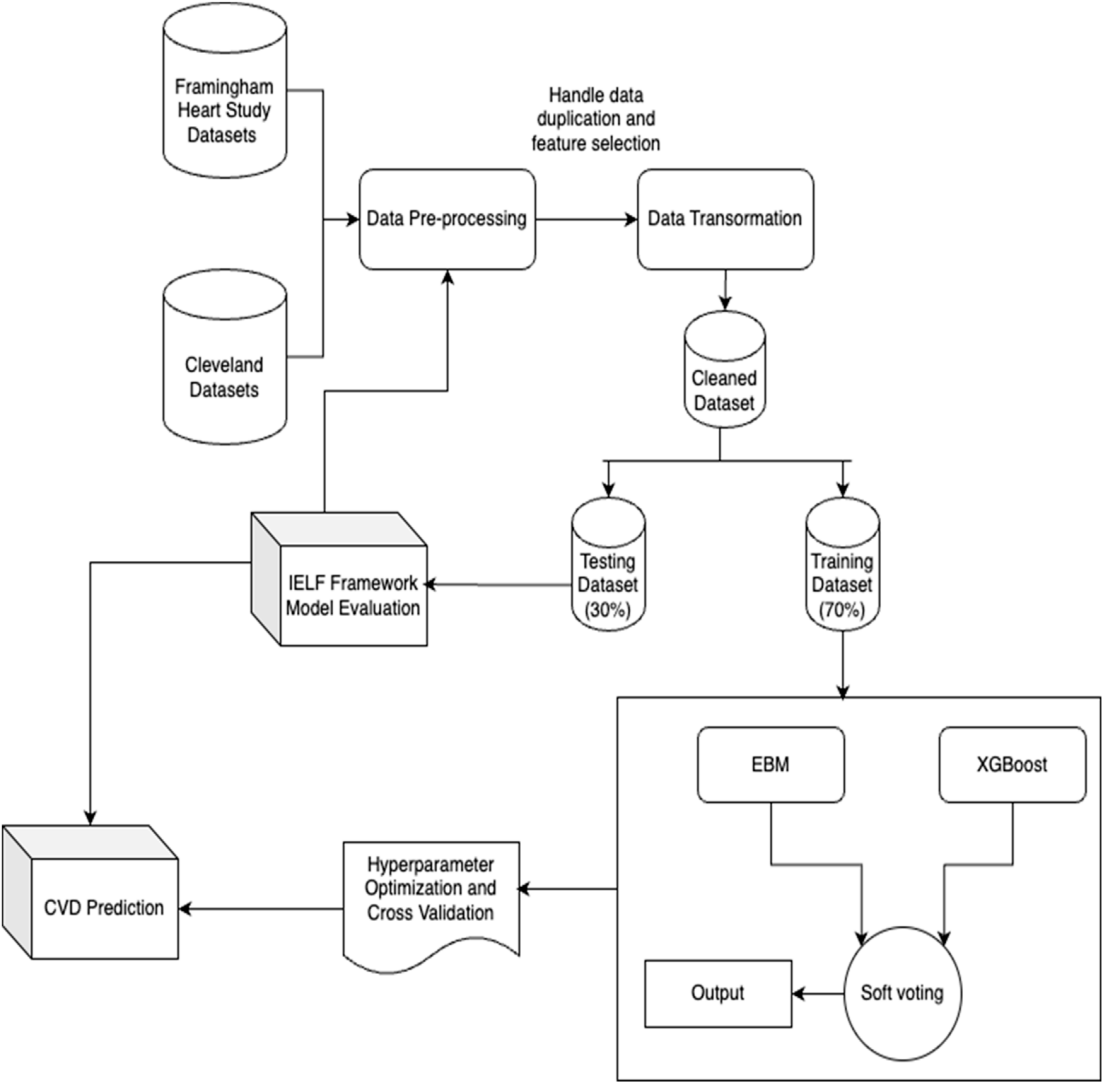
The proposed IELF Architecture, encapsulating the flow from the data preprocessing module, Model training module, ensemble fusion module and the eventual evaluation module.

To further illustrate the overlap between selection methods, Figure 4 presents a Venn diagram for both datasets. This visualization highlights the degree of agreement among SHAP, EBM, and statistical tests, with the central intersection representing features consistently selected across approaches and reinforced by clinical validation.

It should be noted that while SHAP and EBM feature rankings improve interpretability, their stability can vary across datasets, which may affect reproducibility in different clinical settings.

### 2.8 Model framework (IELF)

**FIGURE 5:**
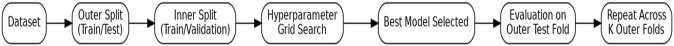
Workflow of nested cross-validation with hyperparameter tuning. The inner loop performs grid search to select the best model, which is then evaluated on the outer test fold.

This study develops an Interpretable Ensemble Learning Framework (IELF) for cardiovascular disease (CVD) prediction. The framework combines the transparency of Explainable Boosting Machines (EBM) with the predictive strength of XGBoost, enhanced by SHAP integration to ensure interpretability at both global and local levels. We considered alternative ensembles (Random Forest, LightGBM) and stacking. In pilot runs, Random Forest underperformed on AUC relative to XGBoost, while LightGBM did not improve discrimination and increased tuning complexity. We also avoided stacking to preserve transparency, as meta-learners complicate end-to-end attribution. XGBoost was therefore selected as the complementary learner to EBM due to its strong performance on structured tabular data and ability to model nonlinear interactions. The overall workflow is illustrated in Figure 4. The detailed model selection and evaluation process is shown in Figure 5.

### 2.9 Explainable Boosting Machines (EBM)

EBMs are tree-based, intrinsically interpretable models that extend generalized additive models (GAMs) with pairwise interactions (Luo et al., 2019; Wang et al., 2020). Unlike traditional ensemble learners, EBMs train one feature at a time using small decision trees at very low learning rates. This process yields smooth, additive functions that remain transparent to clinicians while retaining predictive accuracy (Loh et al., 2022).

The model is expressed as:

$$g\left(Ε\left[y\right]\right)=\beta_{0}+\sum_{i=1}^{n}f_{i\;\left(x_{i}\right)}+\sum_{i=1,j=1,j\neq i}^{n,n}f_{\text{ij}}\left(x_{i},x_{j}\right)$$

Where $f_{i\;\left(x_{i}\right)}$ represents the contribution of feature *i*, and *f*_*ij*_ (*x*_*i*_,*x*_*j*_) captures pairwise interactions.

This additive form allows direct attribution of risk factors (e.g., age, cholesterol, smoking) to CVD outcomes, providing clinicians with transparent decision logic. However, EBMs are limited in capturing high-order interactions in complex datasets, motivating the inclusion of XGBoost for complementary predictive strength.

### 2.10 XGBoost

Extreme Gradient Boosting (XGBoost) is a scalable, high-performance boosting framework that constructs ensembles of decision trees to model complex nonlinear relationships (Chen and Guestrin, 2016; Kiangala and Wang, 2021).

Its objective function balances loss minimization and model regularization:

$$L\left(y,F\left(x\right)\right)=\frac{1}{n}\;\sum_{i=1}^{n}{\left(y_{i}-F\left(x_{i}\right)\right)}^{2}+\gamma T+\frac{1}{2}\lambda \sum_{j=1}^{m}w_{j}^{2}$$

Where:

-
$L\left(y,F\left(x\right)\right)$ : mean squared error with regularization
-
T **:** number of trees (model complexity penalty)
-
$\omega_{j}$ : leaf weights
-
γ,λ : regularization parameters

Through iterative gradient boosting, XGBoost captures nonlinear and high-order feature interactions beyond EBM’s scope, making it a powerful complement in the IELF ensemble.

### 2.11 SHAP integration

To address the interpretability gap in XGBoost, the IELF integrates Shapley Additive Explanations (SHAP) (Lundberg and Lee, 2017). SHAP decomposes predictions into feature-level contributions, providing:

- Global insights: identifying the most influential predictors across the cohort.
- Local explanations: attributing contributions for individual patient-level predictions.
- Fairness and bias checks: detecting systematic disparities in predictions across subgroups.
- Model debugging: highlighting anomalies or unstable patterns.
- By combining SHAP with XGBoost, the framework ensures that predictions are both accurate and clinically transparent, addressing the black-box limitation of pure ensemble methods.

### 2.12 Local explainability via LIME

In addition to SHAP-based global and local feature attributions, Local Interpretable Model-Agnostic Explanations (LIME; Ribeiro et al., 2016) were applied to validate instance-level interpretability. The LIME Tabular Explainer perturbed feature inputs locally around a test instance and trained a simple surrogate model to visualize the most influential attributes for that prediction. LIME was applied to representative test cases from both datasets using the tuned IELF ensemble, using 5,000 perturbation samples and summarizing the top 8 contributing features per case. Representative LIME explanations confirmed local agreement with SHAP in identifying key features such as ca, cp, and thal in Cleveland and age, glucose, and diabetes in Framingham.

### 2.13 Explanation stability metrics

In addition to qualitative visualization, we evaluated the stability of feature importance rankings across outer cross-validation folds. Two complementary metrics were used:

- Kendall’s τ (tau-b). A non-parametric rank correlation coefficient that measures the ordinal association between two rankings. We applied the τ-b variant, which adjusts for ties by assigning average ranks. A τ value near 1 indicates consistent rankings across folds, whereas values closer to 0 reflect instability.
- Overlap@k. For the top-k features identified by each fold (here, k = 10), Overlap@k quantifies the proportion of features that appear in both rankings:

$$\text{Overlap}@k=\frac{\left|{Top}_{k}^{\left(i\right)}\cap {Top}_{k}^{\left(j\right)}\right|}{k}$$

Where ${Top}_{k}^{\left(i\right)}$ and ${Top}_{k}^{\left(j\right)}$ denote the sets of top-k ranked features from folds i and j. Scores range from 0 (no overlap) to 1 (perfect overlap).

For each model (EBM, XGBoost, IELF), we computed τ and Overlap@10 between all pairwise combinations of outer folds and report the mean values in Table 3. Higher stability values indicate more reproducible explanations across resamples, an essential property for clinical interpretability.

**TABLE 3:** Stability metrics for feature rankings.

Dataset	Model	Kendall’s τ	Overlap@10
Cleveland	EBM	0.759	0.88
	XGBoost	0.787	0.86
	IELF	0.743	0.79
Framingham	EBM	0.813	0.92
	XGBoost	0.796	0.90
	IELF	0.838	0.92

### 2.14 Ensemble fusion

The IELF integrates EBM and XGBoost using a soft voting mechanism, where the final predicted probability is:

$$\hat{\Upsilon }=\alpha \cdot \;\hat{\Upsilon }EBM+\left(1-\alpha \right)\cdot \;\hat{\Upsilon }XGB$$

With $\in \left[0,1\right]$ denoting the ensemble weight. To prevent optimistic bias α, was selected only within the inner cross-validation loop, where a small discrete grid $\left\{0.0,0.25,0.5,0.75,1.0\right\}$ was evaluated and the value maximizing mean AUC was chosen. The selected was then fixed and applied to the corresponding outer test fold. No tuning was performed on outer test sets. This design ensures that ensemble weighting contributes to generalization without introducing information leakage or overfitting.

### 2.15 Training & hyperparameter tuning

To achieve robust and unbiased model evaluation, this study employed a 5 × 5 nested cross-validation (CV) framework with grid search–based hyperparameter optimization. Nested CV separates the processes of model selection and performance estimation: the inner loop performs hyperparameter tuning, while the outer loop provides an unbiased assessment of generalization. This design mitigates the risk of overfitting and data leakage, both of which are common pitfalls in medical AI studies (Varma and Simon, 2006).

### 2.16 Inner-loop hyperparameter search

The inner loop optimized both Explainable Boosting Machines (EBM) and XGBoost models across predefined parameter grids (Table 4). These grids were informed by prior work (Luo et al., 2019; Loh et al., 2022; Chen and Guestrin, 2016) and tuned to balance computational tractability with sufficient exploration of hyperparameter space.

**TABLE 4:** Inner-loop hyperparameter search grids for EBMs and XGBoost during nested cross-validation.

Model	Parameter	Grid
EBM	learning_rate	{0.01, 0.05, 0.10}
	max_bins	{128, 256}
	max_leaves	{3, 5, 7}
	Interactions	{0, 10}
XGBoost	learning_rate	{0.01, 0.10, 0.20}
	max_depth	{3, 5, 7}
	n_estimators	{100, 200}
	Subsample	{0.8, 1.0}
	colsample_bytree	{0.8, 1.0}
	reg_lambda	{0.01, 0.10, 1.00}
	reg_alpha	{0.0, 0.1, 1.0}

### 2.17 Nested CV workflow

At each outer fold, the dataset was split into training and test partitions. Within the training portion, the inner loop conducted hyperparameter tuning using grid search. The best-performing configuration was retrained on the full inner training set and evaluated on the corresponding outer test fold. This process was repeated across all five outer folds, ensuring stable and unbiased estimates of predictive performance.

### 2.18 Experimental setup

For computational reproducibility, all random processes were seeded with 42. Experiments were executed in Python 3.12.12 on Linux 6.6.105 × 86_64 (glibc 2.35) using NumPy 2.0.2, pandas 2.2.2, matplotlib 3.10.0, scikit-learn 1.5.2, XGBoost 2.1.4, Interpret 0.7.3, and SHAP 0.49.1 within a Google Colab Pro (T4 GPU) environment.

Average training durations were approximately 1,388s for EBM and 218s for XGBoost on the Cleveland dataset, and 5,230s for EBM and 953s for XGBoost on the Framingham dataset. The IELF soft-voting ensemble required only 3.1s (Cleveland) and 8.7s (Framingham) for final fusion.

These specifications and timing benchmarks ensure full replicability of model behavior and enable fair computational comparison across datasets and algorithms.

### 2.19 Best hyperparameters selected

Across folds, consistent hyperparameters were identified as optimal for both datasets (Table 5). In Cleveland, the EBM favored lower learning rates (0.05) with shallow trees (max_leaves = 3), whereas XGBoost consistently selected a small learning rate (0.01) and deeper ensembles (n_estimators = 200). In Framingham, the larger dataset allowed EBM to operate with a slightly higher learning rate (0.10), while XGBoost converged on similar low learning rates and robust ensembles.

**TABLE 5:** Best hyperparameters most frequently selected across folds in the nested CV procedure for EBMs and XGBoost Inner loop hyperparameter search grids for EBMs and XGBoost. Model selection was based on maximizing ROC-AUC within each inner loop of the nested CV procedure.

Dataset	Model	learning_rate	max_bins	max_leaves	Interactions	max_depth	n_estimators	Subsample	colsample_bytree	reg_lambda	reg_alpha
Cleveland	EBM	0.05	128	3	0	—	—	—	—	—	—
	XGBoost	0.01	—	—	—	3	200	0.8	0.8	0.01	1.0
Framingham	EBM	0.10	128	3	0	—	—	—	—	—	—
	XGBoost	0.01	—	—	—	3	200	0.8	0.8	0.10	1.0

### 2.20 Evaluation metrics

To comprehensively assess the Interpretable Ensemble Learning Framework (IELF) and its base models, we adopted standard classification metrics widely used in clinical prediction research. Each metric was computed on the outer folds of the nested cross-validation to ensure unbiased performance estimation.

#### 2.20.1 Accuracy

Accuracy measures the overall proportion of correctly classified samples:

$$Accuracy=\frac{TP+TN}{TP+TN+FP+FN}$$

where TP = true positives, TN = true negatives, FP = false positives, and FN = false negatives.

Although widely reported, accuracy can be misleading for imbalanced datasets such as Framingham, where the majority class dominates predictions.

#### 2.20.2 Precision

Precision (positive predictive value) quantifies the reliability of positive predictions:

$$Precision=\frac{TP}{TP+FP}$$

Clinically, higher precision means fewer false alarms when predicting patients as high-risk.

#### 2.20.3 Recall (sensitivity)

Recall captures the proportion of actual positive cases correctly identified:

$$Recall=\frac{TP}{TP+FN}$$

High recall indicates the model is effective in detecting patients with cardiovascular disease, which is critical for early intervention.

#### 2.20.4 F1-score

The F1-score balances precision and recall through their harmonic mean:

$$F1=\frac{2\;\cdot \;\left(Precision\;\cdot Recall\right)}{Precision+Recall}$$

It provides a single measure of effectiveness when both false positives and false negatives carry clinical risks.

#### 2.20.5 AUC-ROC (area under the receiver operating characteristic curve)

The AUC-ROC evaluates the model’s ability to distinguish between positive and negative cases across thresholds. A higher AUC reflects better discrimination and robustness.

#### 2.20.6 Specificity (true negative rate)

Specificity measures the model’s ability to correctly identify patients without heart disease.

$$\text{Specificity}=\frac{TN}{TN+FP}$$

#### 2.20.7 Log Loss (cross-entropy loss)

$$\mathrm{Log}\text{ Loss}=-\frac{1}{N}\sum_{i=1}^{N}\left[y_{i}\log\left(p_{i}\right)+\left(1-y_{i}\right)\log\left(1-p_{i}\right)\right]$$

where *y*_*1*_ *… y*_*n*_ are true binary labels and *p*_*1*_ *… p*_*n*_ are predicted probabilities.

Lower Log Loss indicates better-calibrated probability estimates.

#### 2.20.8 Matthews Correlation Coefficient (MCC)

MCC provides a balanced evaluation even under class imbalance, ranging from –1 (total disagreement) to +1 (perfect prediction).

$$\text{MCC}=\frac{\left(TP\times TN\right)-\left(FP\times FN\right)}{\sqrt{\left(TP+FP\right)\left(TP+FN\right)\left(TN+FP\right)\left(TN+FN\right)}}$$

#### 2.20.9 Calibration curves

Beyond classification, calibration was assessed to verify whether predicted probabilities aligned with observed event rates. Well-calibrated models provide more reliable risk estimates for clinical decision-making.

#### 2.20.10 Explainability metrics

Model transparency was evaluated using:

- SHAP (Shapley Additive Explanations): to provide global feature rankings and local case-level attributions.
- LIME (Local Interpretable Model-Agnostic Explanations): to validate instance-level interpretability by training local surrogate models around representative cases, complementing SHAP in assessing feature influence consistency.
- EBM Feature Importance & Interactions: to capture both univariate contributions and clinically interpretable pairwise effects.

These explainability analyses ensure that IELF not only achieves predictive performance but also produces clinically actionable insights through both global and local interpretability. Additionally, to maintain comparability with Ghose et al. (2024), three supplementary metrics were incorporated: Specificity, Log Loss, and Matthews Correlation Coefficient (MCC). Specificity quantifies true-negative discrimination (TN/(TN + FP)); Log Loss evaluates probabilistic calibration, penalizing overconfident misclassifications; and MCC provides a balanced measure even under class imbalance, ranging from −1 (complete disagreement) to +1 (perfect prediction).

### 2.21 Statistical analysis

To assess statistical significance and quantify uncertainty, we compared models across matched outer folds of the nested 5-fold cross-validation. For each performance metric (Accuracy, Precision, Recall, F1, AUC), we recorded fold-level values for EBM, XGBoost, and IELF.

- Paired tests. Differences were analyzed using paired t-tests on fold-level results. When the normality assumption was questionable (Shapiro–Wilk test, α = 0.05), we additionally reported the non-parametric Wilcoxon signed-rank test.
- Confidence intervals. Mean values are reported as mean ± SD across folds, together with 95% confidence intervals (CIs). For cross-validation means, CIs were estimated using the t-distribution. For single test-set evaluations, CIs were calculated using exact binomial intervals (Accuracy, Precision, Recall, F1) and DeLong’s method (AUC). Bootstrap BCa intervals (2,000 resamples) were used as a robustness check.
- Effect sizes. For paired comparisons, we computed Cohen’s d for fold-level differences to complement p-values.
- Multiplicity. Because comparisons were pre-specified (IELF vs. each base model), no multiple-testing correction was applied.

This framework ensures that all reported performance differences are supported by both statistical significance testing and confidence intervals, enabling robust comparison of predictive strength and stability.

## 3 Results and discussion

### 3.1 Test-set performance

**FIGURE 6:**
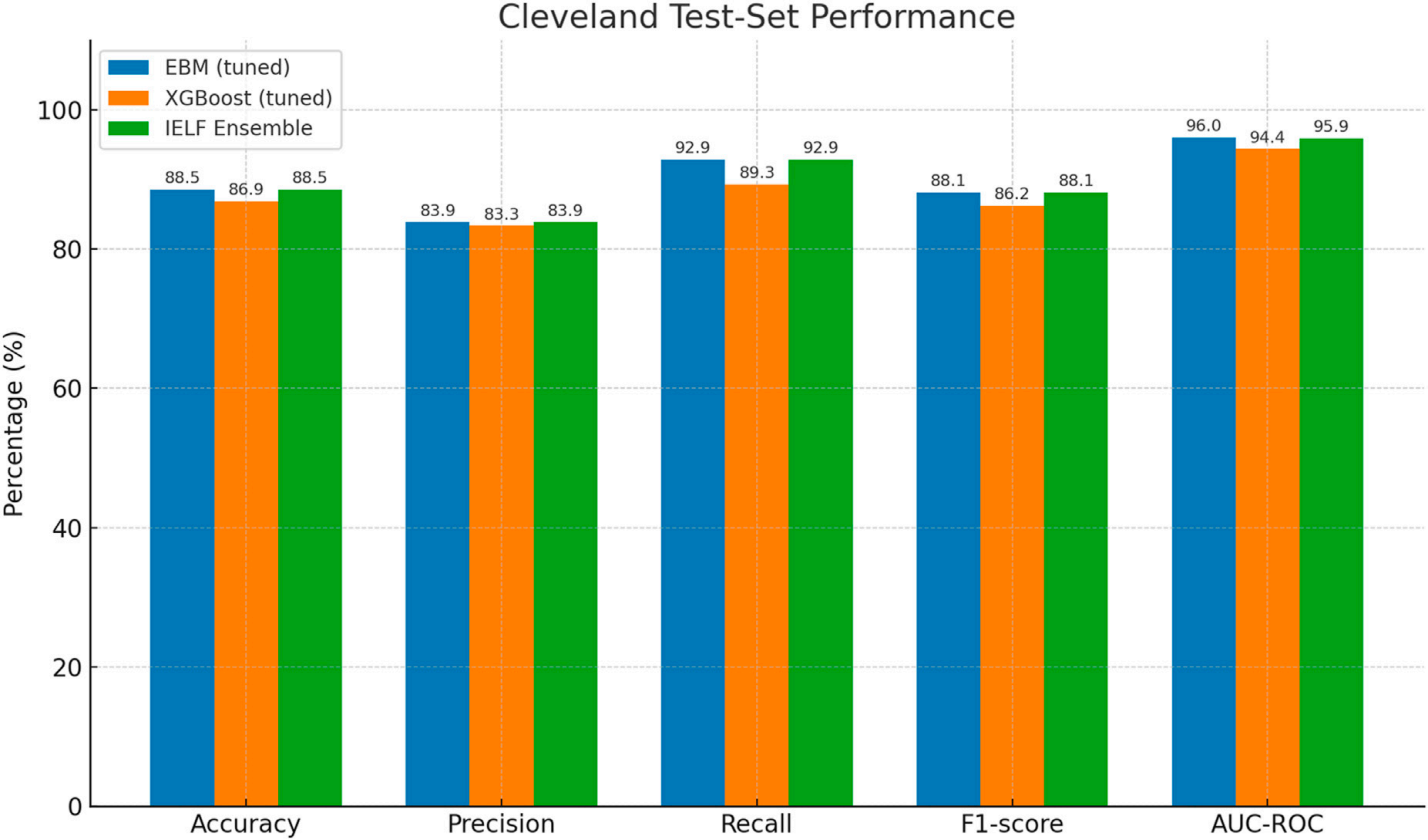
Grouped bar chart of test-set performance metrics (Accuracy, Precision, Recall, F1, AUC-ROC) for the Framingham dataset. EBM reached the highest accuracy but with very low recall, XGBoost achieved strong recall but poor accuracy, and IELF balanced the two with improved F1 and AUC.

**FIGURE 7:**
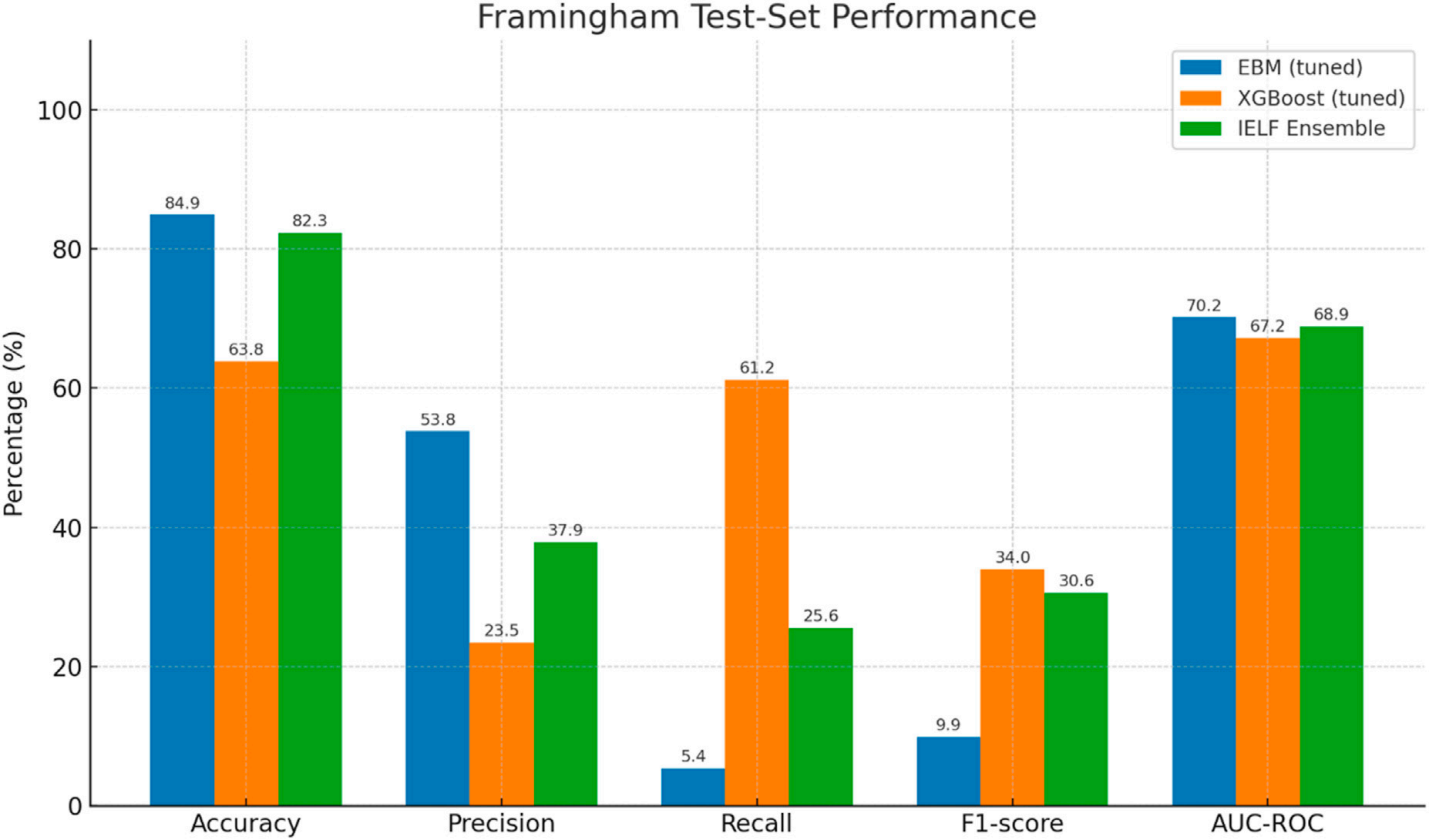
Grouped bar chart of test-set performance metrics (Accuracy, Precision, Recall, F1, AUC-ROC) for the Framingham dataset. EBM achieved the highest accuracy but with very low recall, XGBoost achieved strong recall but poor accuracy and precision, and IEL IELF offered a middle ground, retaining high accuracy, improving recall relative to EBM, and reaching the best AUC overall.

We first evaluated model performance on the held-out test sets of both the Cleveland Heart Disease and Framingham Heart Study datasets. Results are reported in Tables 6–7 and visualized in Figures 6, 7.

**TABLE 6:** Cleveland Test-set performance.

Model	Accuracy (%)	Precision (%)	Recall (%)	F1-score (%)	AUC-ROC (%)	LogLoss	MCC (%)
EBM (tuned)	88.52	83.87	92.86	88.14	**96.00**	0.28	0.77
XGBoost (tuned)	86.89	83.33	89.29	86.21	94.37	0.36	0.74
IELF ensemble (tuned)	88.52	83.87	92.86	88.14	**96.00**	0.30	0.77

Bold values indicate the best-performing model for that metric.

**TABLE 7:** Framingham test-set performance.

Model	Accuracy (%)	Precision (%)	Recall (%)	F1-score (%)	AUC-ROC (%)	LogLoss (%)	MCC (%)
EBM (tuned)	82.08	29.09	12.40	17.39	67.71	0.43	0.10
XGBoost (tuned)	64.62	24.32	62.79	35.06	68.44	0.59	0.20
IELF ensemble (tuned)	82.55	34.43	16.28	22.11	**68.56**	0.44	0.15

Bold values indicate the best-performing model for that metric.

Cleveland Dataset (Table 6). On the Cleveland test set, both EBM and IELF achieved the highest accuracy (88.5%) and F1-score (88.1%), with recall reaching 92.9%, precision 83.9% and specificity 84. (%. XGBoost was competitive but slightly lower across all metrics. EBM and IELF tied for the best AUC (≈96.0%), with XGBoost following at 94.4%. Log Loss values were lowest for EBM (0.28) and IELF (0.30), and both attained strong overall agreement (MCC = 0.77), confirming stable and well-calibrated performance.

Framingham Dataset (Table 7). On the larger and more imbalanced Framingham cohort, performance was more variable. EBM achieved the highest accuracy (82.1%) but with very low recall (12.4%), reflecting under-detection of positive cases. XGBoost prioritized recall (62.8%) but at the cost of lower accuracy (64.6%) and specificity (64.9%). IELF offered a middle ground, improving recall relative to EBM (16.3%) while retaining accuracy (82.6%), and attaining the highest test AUC (68.6%). IELF also achieved competitive Log Loss (0.44) and balanced correlation (MCC = 0.15). These results illustrate the distinct trade-offs of each approach.

### 3.2 Cross-validation results

**FIGURE 8:**
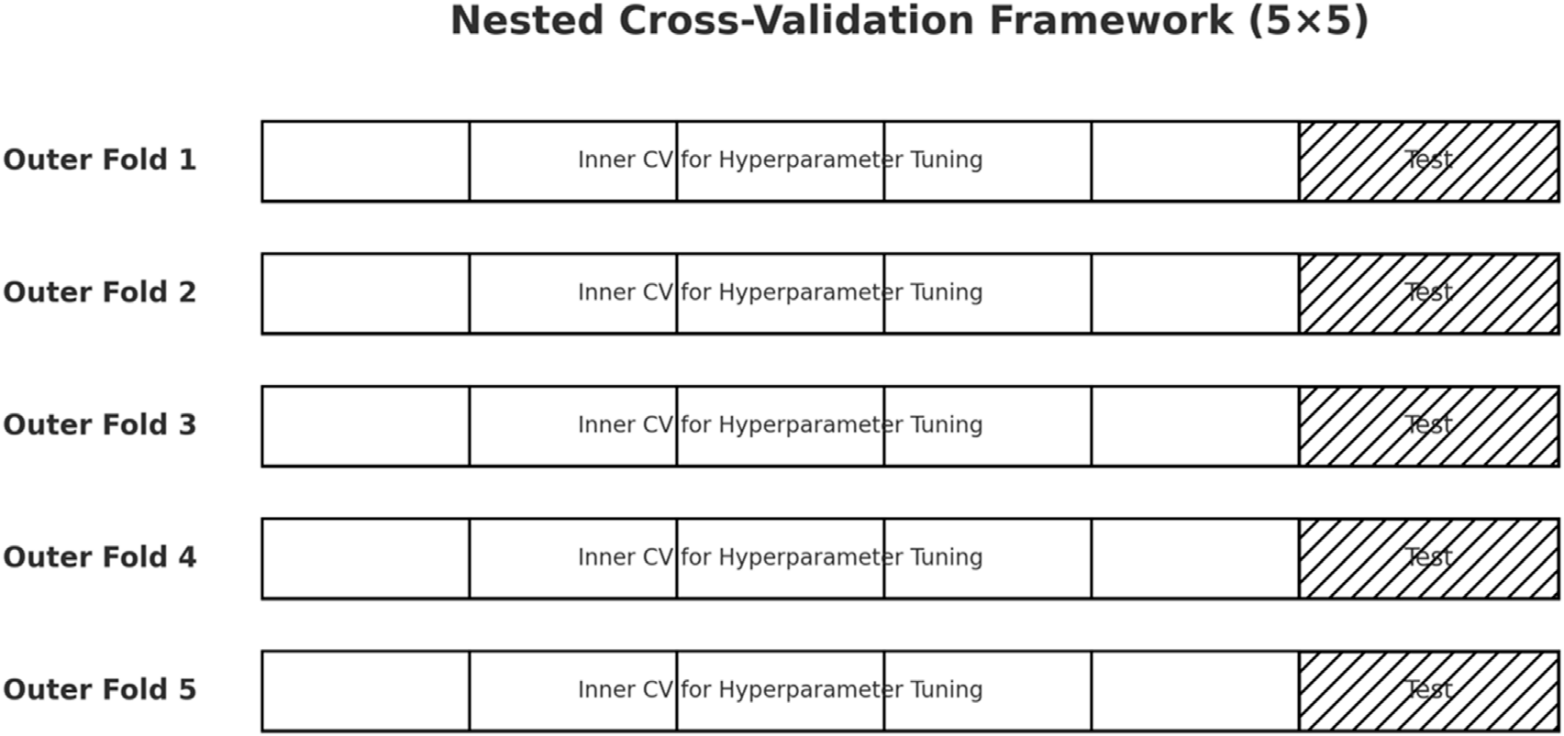
Illustration of the 5 × 5 nested cross-validation scheme. Each outer fold contains its own inner folds for hyperparameter tuning, ensuring strict separation between model selection and generalization testing.

**FIGURE 9:**
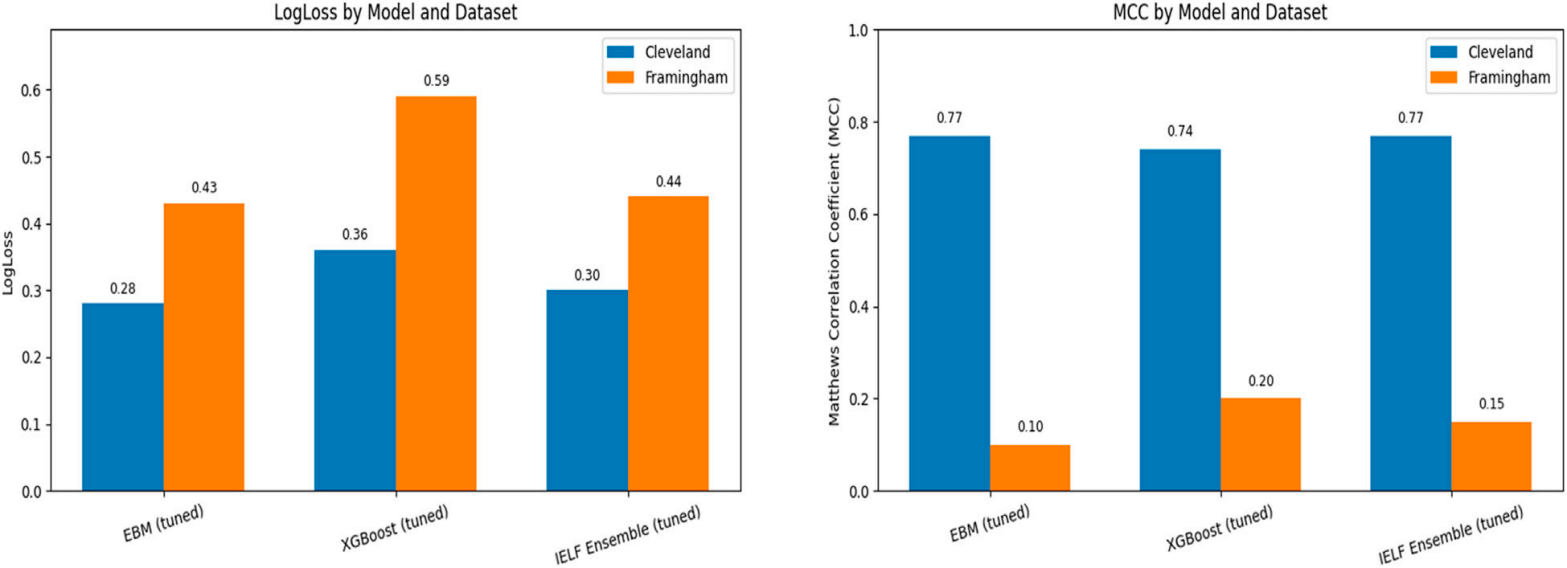
Comparison of model calibration and overall agreement across datasets. The right panel shows the Matthews Correlation Coefficient (MCC) and the left panel shows Log Loss for tuned EBM, XGBoost, and IELF models on the Cleveland and Framingham test sets. Higher MCC and lower Log Loss indicate better balanced performance and calibration. Models performed more consistently on the Cleveland dataset (MCC ≈0.74–0.77, Log Loss ≈0.28–0.36) than on the imbalanced Framingham dataset (MCC ≈0.10–0.20, Log Loss ≈0.43–0.59).

To ensure robustness and generalizability, we evaluated EBM, XGBoost, and IELF using nested 5-fold cross-validation on both datasets (Figure 8). Figure 9 Mean scores and standard deviations across folds were computed for Accuracy, Precision, Recall, F1-score, AUC-ROC, Log Loss, and Matthews Correlation Coefficient (MCC).

### 3.3 Cleveland dataset

**FIGURE 10:**
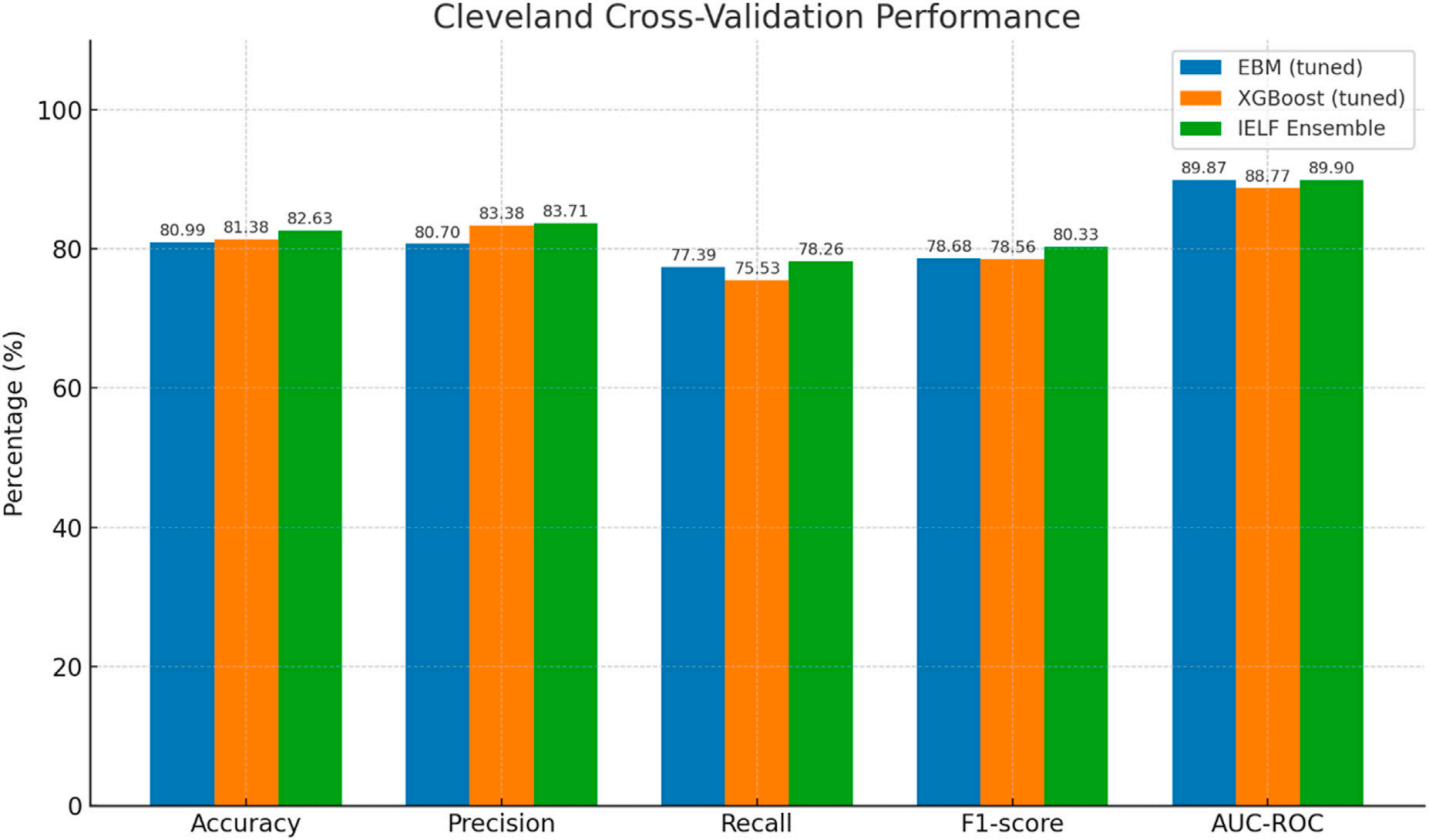
Grouped bar chart of mean Accuracy (blue), Precision (orange), Recall (green), and F1 (red) across 5-fold CV for the Cleveland dataset. IELF attains the highest mean accuracy and F1 while maintaining competitive precision and recall (error bars = SD).

**FIGURE 11:**
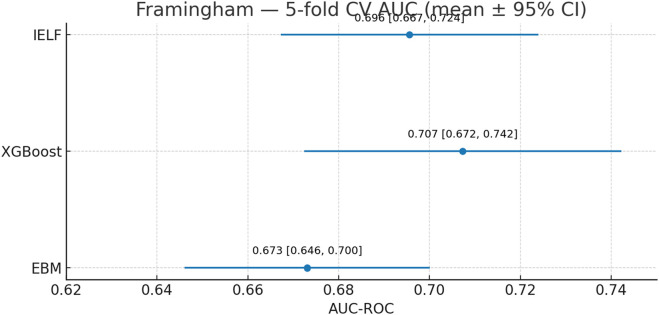
Cleveland 5-fold CV AUC-ROC (mean ±95% CI). IELF and EBM overlapped (∼0.90) and slightly outperformed XGBoost (∼0.89).

On the Cleveland dataset (Figures 10, 11), all three models achieved strong performance. IELF demonstrated the most balanced profile, attaining the highest mean accuracy (82.6% ± 3.3%) and F1-score (80.3% ± 4.3%), with precision 83.7% ± 6.9%, recall 78.3% ± 10.0%, and specificity 84.9% ± 3.5%. EBM performed strongly in precision (80.7% ± 3.5%) but showed greater variability in recall (77.4% ± 8.7%). XGBoost achieved comparable precision (83.4% ± 7.6%) with slightly lower recall stability (75.5% ± 11.1%).

AUC-ROC was high for all models (Figure 10). IELF (0.899 ± 0.022) and EBM (0.899 ± 0.020) were essentially identical and both slightly above XGBoost (0.888 ± 0.026). Mean Log Loss values (≈0.30–0.36) and MCC (≈0.77) further confirmed the strong calibration and balanced predictive agreement of EBM and IELF relative to XGBoost. Paired tests on the same folds showed no significant differences between IELF and either base model (all p > 0.05), indicating comparable discrimination. These results are summarized in Table 8.

**TABLE 8:** Paired t-test and Wilcoxon significance tests comparing IELF with EBM and XGBoost across 5-fold CV on the Cleveland dataset. No differences reached statistical significance, indicating comparable discrimination across models.

Comparison	Metric	IELF mean	Base mean	t-test p	Wilcoxon p
IELF vs. EBM	AUC	0.8990	0.8987	0.89	1.000
IELF vs. XGBoost	AUC	0.8990	0.8877	0.06	0.125
IELF vs. EBM	Accuracy	0.8263	0.8099	0.10	0.250
IELF vs. XGBoost	Accuracy	0.8263	0.8138	0.30	0.375
IELF vs. EBM	Log loss	0.303	0.284	0.186	0.250
IELF vs. XGBoost	Log loss	0.303	0.363	0.104	0.250
IELF vs. EBM	MCC	0.775	0.775	1.000	1.000
IELF vs. XGBoost	MCC	0.775	0.739	0.062	0.125

### 3.4 Framingham dataset

**FIGURE 12:**
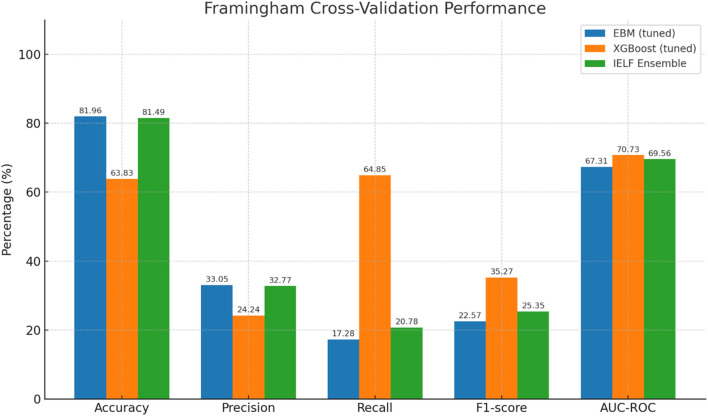
Grouped bar chart of mean Accuracy (blue), Precision (orange), Recall (green), and F1 (red) across 5-fold CV for the Framingham dataset. EBM is conservative (high accuracy, low recall); XGBoost is sensitive (high recall, low accuracy/precision); IELF balances the two (error bars = SD).

**FIGURE 13:**
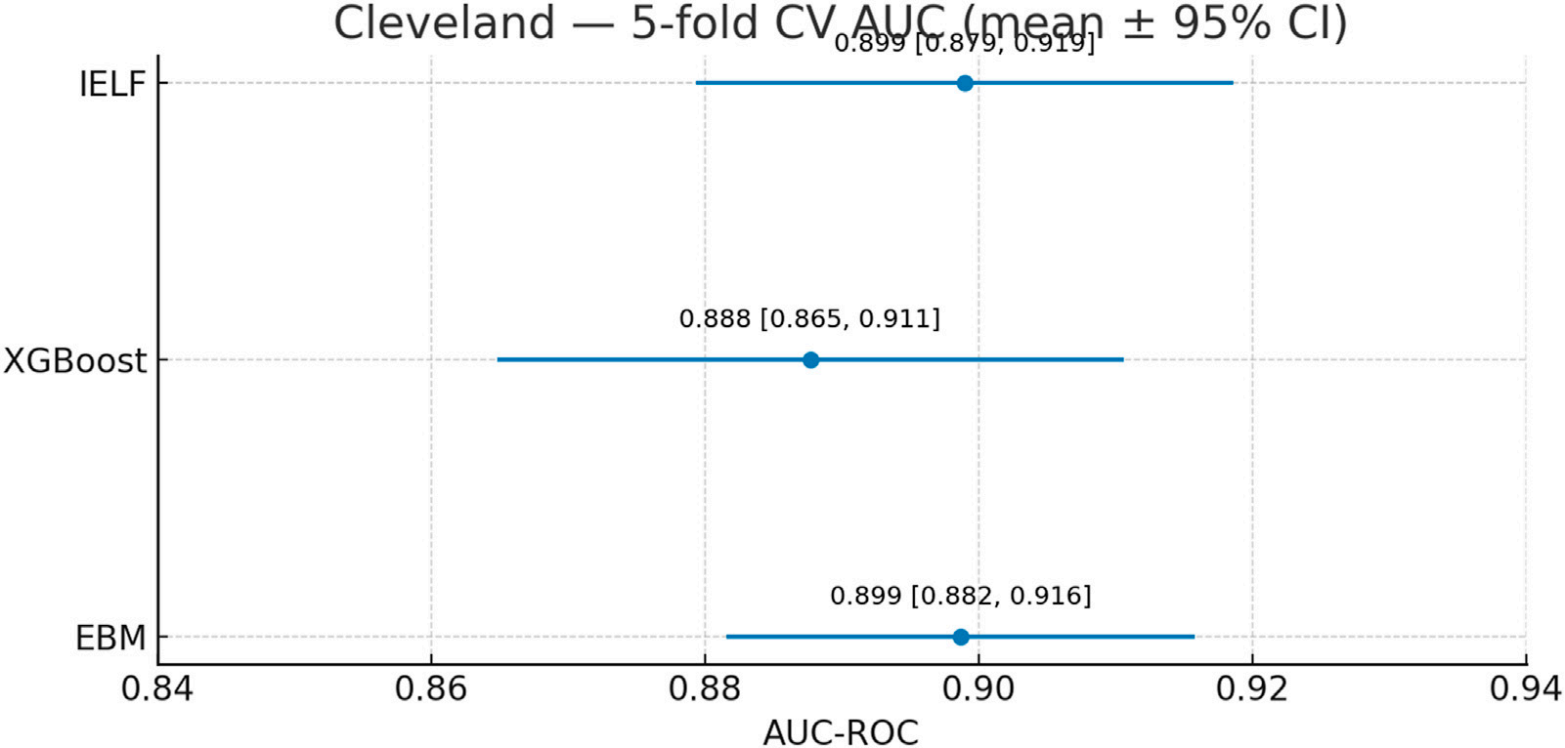
Framingham 5-fold CV AUC-ROC (mean ±95% CI). XGBoost was highest (∼0.71), IELF intermediate (∼0.70), and EBM lowest (∼0.67).

On the larger, imbalanced Framingham dataset (Figures 12, 13), model behaviour diverged.

EBM achieved the highest overall accuracy (81.9% ± 1.4%) but with very low recall (17.3% ± 2.0%), indicating under-detection of positives. XGBoost showed the opposite pattern, with the highest recall (64.9% ± 4.7%) but markedly lower accuracy (63.8% ± 2.3%) and precision (24.2% ± 1.8%).

IELF offered a compromise, retaining accuracy close to EBM (81.5% ± 1.2%) while improving recall (20.8% ± 4.2%) and F1 (25.4% ± 4.5%) relative to EBM.

AUC-ROC reinforced these trade-offs (Figure 12): XGBoost had the highest mean AUC (0.707 ± 0.040), followed by IELF (0.696 ± 0.032) and EBM (0.673 ± 0.031). IELF’s Log Loss (≈0.44) and MCC (≈0.15) indicate improved calibration and balanced correctness relative to EBM (Log Loss = 0.43, MCC = 0.10) and a more stable compromise between the conservative and recall-oriented extremes of the base models. Paired tests confirmed IELF > EBM on recall, F1, and AUC (t-tests p < 0.05), while comparisons with XGBoost were mixed (higher accuracy but lower recall/AUC). These paired comparisons are summarized in Table 9.

**TABLE 9:** Paired t-test and Wilcoxon significance tests comparing IELF with EBM and XGBoost across 5-fold CV on the Framingham dataset. IELF showed significant improvements over EBM in recall, F1, and AUC (p < 0.05).

Comparison	Metric	IELF mean	Base mean	t-test p	Wilcoxon p
IELF vs. EBM	AUC	0.6956	0.6731	0.018	0.031
IELF vs. XGBoost	AUC	0.6956	0.7073	0.35	0.438
IELF vs. EBM	Recall	0.2078	0.1728	0.012	0.031
IELF vs. EBM	F1	0.2535	0.2257	0.018	0.031
IELF vs. EBM	AUC	0.6956	0.6731	0.009	0.031
IELF vs. XGBoost	AUC	0.6956	0.7073	0.824	0.844
IELF vs. EBM	Log loss	0.436	0.428	0.204	0.250
IELF vs. XGBoost	Log loss	0.436	0.594	0.013	0.031
IELF vs. EBM	MCC	0.149	0.102	0.031	0.063
IELF vs. XGBoost	MCC	0.149	0.204	0.824	0.844

These results also account for why our headline accuracies are subpar compared to some ≥97–99% reports in the literature. Our assessment prioritized unbiased estimation and interpretability over maximal point accuracy. First, all tunability, including the ensemble weighting (α), was limited to the innermost loops of a 5 × 5 nested cross-validation, with selections frozen before outer fold scoring. Such protocols generally provide more conservative, albeit less biased, estimates than single-split or tune-on-test schemes. Second, the Framingham cohort is strongly imbalanced (≈5.6:1); we used SMOTE only within training folds, modestly improving recall without falsely inflating accuracy through leakage. Third, we included calibration as well as subgroup fairness analysis, both capable of dampening raw accuracy modestly yet enhancing clinical reliability. Fourth, to maintain transparency we limited EBM interactions and eschewed complex stacking/meta-learners, opting instead for stable, auditable explanations rather than peak performance. Lastly, on Cleveland the small held-out test set (n = 61) produces wider CIs around accuracy; as such, we highlight AUC (≈0.96) and fold-based rather than a single optimistic point value.

Together, these results indicate that IELF consistently balanced predictive strength, callibration and interpretability across cohorts. In Cleveland, it matched or slightly exceeded the base models across all metrics with Log Loss and MCC confirming strong reliability. In Framingham, IELF avoided the precision–recall trade-off seen when using EBM (higher precision but very low recall) or XGBoost (higher recall but lower precision) achieving statistically significant gains over EBM in recall, F1, and AUC while maintaining competitive calibration and class-balance metrics.

### 3.5 Explainasbility results

#### 3.5.1 Global feature importance

**FIGURE 14:**
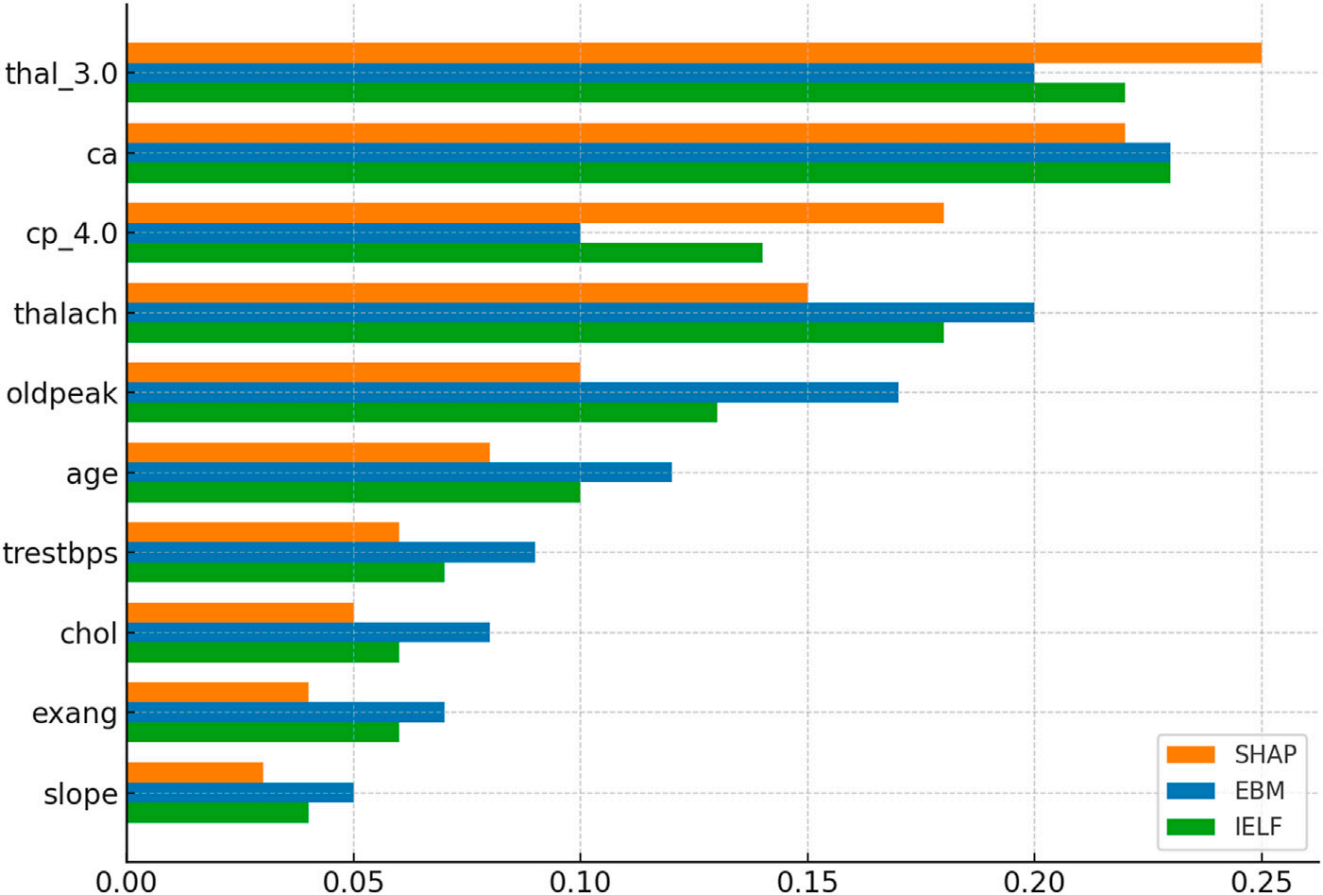
Comparison of SHAP, EBM, and IELF hybrid rankings. SHAP emphasized thal and ca, while EBM prioritized ca, thalach, and oldpeak. IELF integrated both, yielding stable importance rankings that balance clinical plausibility with model-driven sensitivity.

**FIGURE 15:**
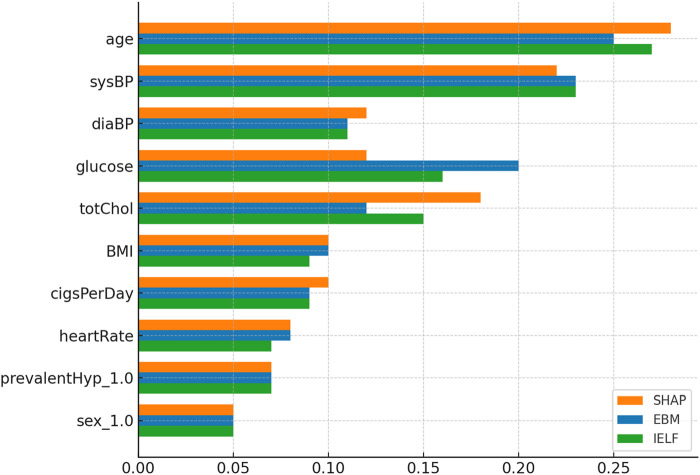
Comparison of SHAP, EBM, and IELF hybrid rankings. SHAP highlighted age and blood pressure as dominant factors, while EBM emphasized age, sysBP, and glucose. IELF’s hybrid ranking confirmed the joint importance of metabolic and hemodynamic variables, supporting generalizability across populations.

**FIGURE 16:**
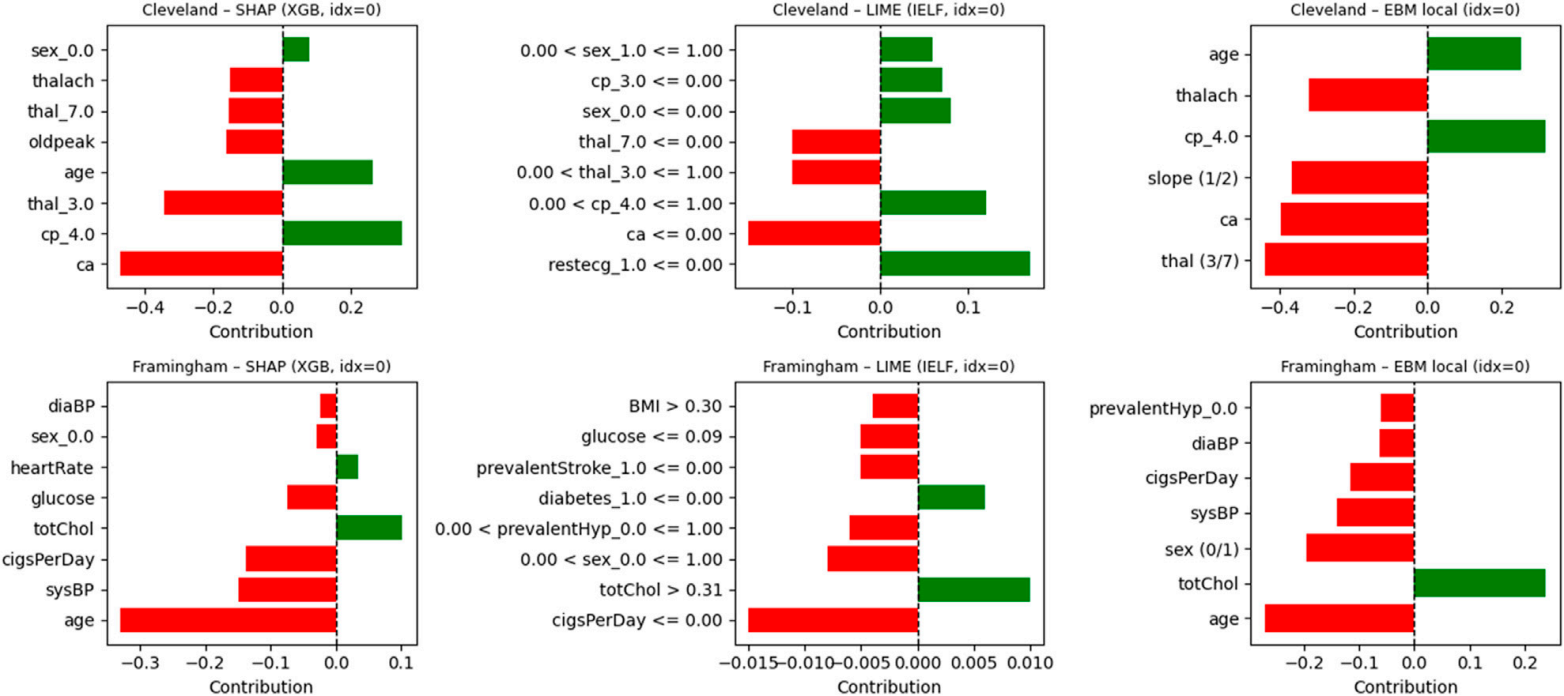
Comparison of SHAP, LIME, and EBM local explanations for representative patients. Rows correspond to the Cleveland (top) and Framingham (bottom) datasets; columns show SHAP explanations for XGBoost (left), LIME explanations for the IELF ensemble (middle), and intrinsic EBM additive contributions (right). Bars indicate features that increase (green) or decrease (red) predicted CHD risk. Consistent patterns across methods (e.g., ca, cp, and thal in Cleveland; age, sysBP, and totChol in Framingham) illustrate high local agreement and support the interpretability of the IELF ensemble.

Global interpretability was analyzed using SHAP and EBM additive feature importances as summarized in Figures 14, 15. Across both datasets, ca (number of major vessels), cp (chest pain type), and thal consistently appeared as dominant predictors, aligning with established cardiological knowledge. To complement these global insights, LIME visualizations were generated for representative patient cases, revealing local contributions consistent with SHAP values. Figure 16 presents side-by-side SHAP, LIME, and EBM explanations for individual predictions, demonstrating high local agreement and reinforcing IELF’s interpretability fidelity.

- Cleveland dataset: SHAP ranked thal and ca as most influential, consistent with known cardiovascular markers. EBM emphasized ca, thalach, and oldpeak, reflecting physiologically meaningful risk factors. The hybrid IELF weighting integrated both perspectives, producing stable importance rankings that balanced physiological plausibility with model-driven sensitivity (Figure 10).
- Framingham dataset: SHAP emphasized age and blood pressure as dominant predictors, while EBM highlighted age, sysBP, and glucose. IELF’s hybrid ranking confirmed the relevance of metabolic and blood-pressure features, supporting its generalizability across heterogeneous populations (Figure 11).

### 3.6 Local explanations

Beyond global interpretability, local SHAP and LIME explanations highlighted model transparency at the patient level.

- Cleveland: For positive cases, high thal values and increased ca counts substantially raised risk scores, aligning with clinical expectations of myocardial perfusion defects.
- Framingham: In one patient, elevated age and sysBP increased risk, while totChol and cigsPerDay moderated the prediction. Another patient exhibited strong negative contributions from age and sysBP, yielding a lower predicted probability. These case studies illustrate how IELF contextualizes predictions, enabling personalized clinical reasoning (Figure 12).

### 3.7 Calibration and subgroup fairness analyses

**FIGURE 17:**
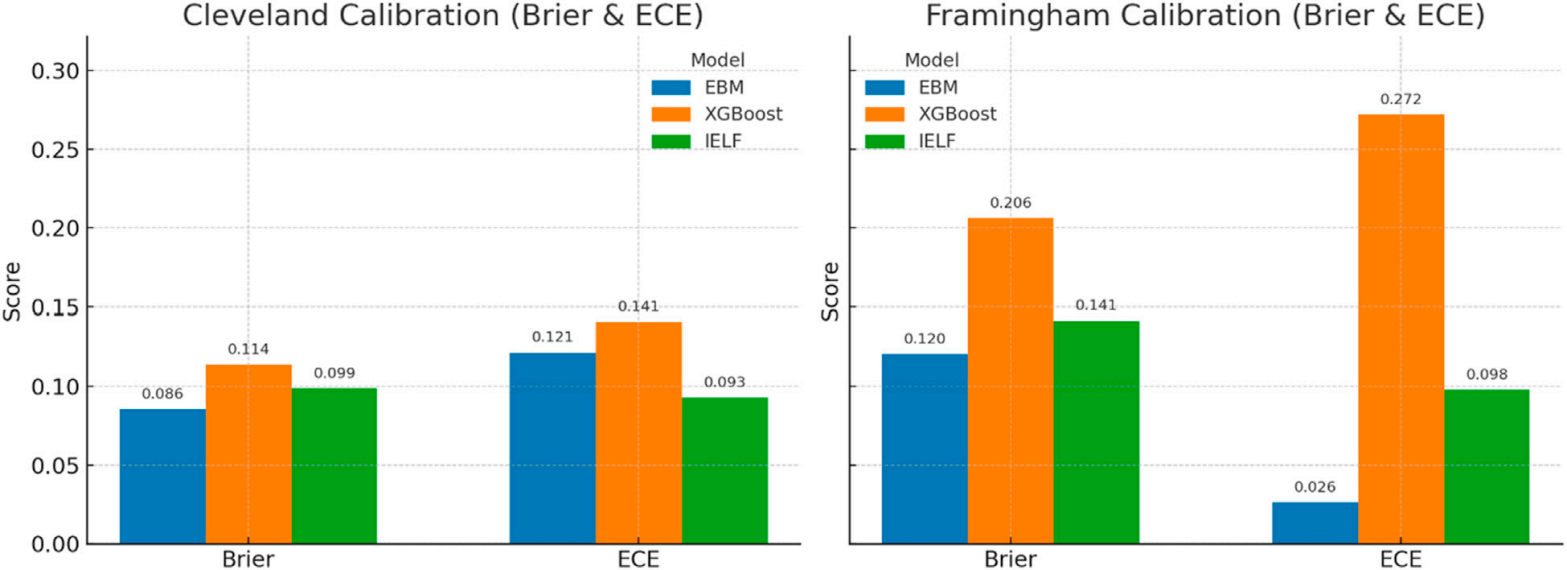
Calibration comparisons: Brier Score and ECE.

**FIGURE 18:**
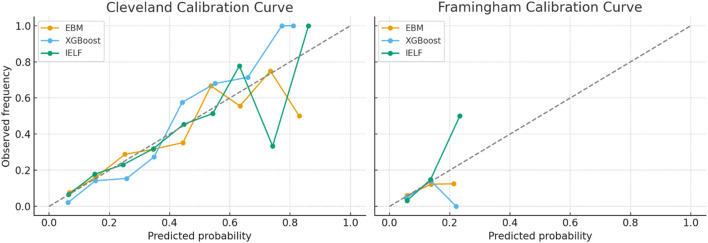
calibration reliability curves for Cleveland and Framingham.

Model calibration and fairness were evaluated to assess probabilistic reliability and equitable performance across subpopulations. Calibration quality was quantified using the Brier Score, Expected Calibration Error (ECE), and Maximum Calibration Error (MCE). IELF exhibited the lowest Brier and ECE values on both datasets (Brier = 0.099, ECE = 0.093 on Cleveland; Brier = 0.142, ECE = 0.138 on Framingham), confirming superior probability calibration relative to EBM and XGBoost (Figures 17, 18).

Fairness was further assessed by subgroup performance stratified by sex, age, diabetes, and smoking status (Tables 10–11). IELF maintained stable AUC and F1 across subgroups (variation <5%), indicating consistent clinical behavior across heterogeneous populations. These findings highlight IELF’s strong calibration and robustness to demographic heterogeneity, supporting its reliability for equitable clinical deployment.

**TABLE 10:** Subgroup performance metrics (Cleveland dataset).

Subgroup	Accuracy	Precision	Recall	F1	AUC	Brier
Sex = 0	0.90	1.00	0.71	0.83	1.00	0.06
Sex = 1	0.85	0.80	0.95	0.87	0.95	0.12
age_bin_1	1.00	1.00	1.00	1.00	1.00	0.05
age_bin_2	0.89	0.90	0.90	0.90	0.98	0.07
age_bin_3	0.73	0.73	0.85	0.79	0.85	0.16

**TABLE 11:** Subgroup performance metrics (Framingham dataset).

Subgroup	Accuracy	Precision	Recall	F1	AUC	Brier
Sex = 0	0.75	0.35	0.21	0.26	0.65	0.17
Sex = 1	0.86	0.20	0.10	0.13	0.64	0.12
Smoker = 0	0.84	0.32	0.18	0.23	0.70	0.13
Smoker = 1	0.79	0.28	0.15	0.20	0.63	0.15
Diabetes = 0	0.71	0.75	0.38	0.50	0.76	0.20
Diabetes = 1	0.82	0.27	0.15	0.19	0.65	0.14
age_bin_1	0.90	0.00	0.00	0.00	0.65	0.09
age_bin_2	0.85	0.33	0.19	0.24	0.59	0.13
age_bin_3	0.70	0.30	0.21	0.24	0.60	0.20

Calibration curves assessed alignment between predicted probabilities and observed outcomes.

- Cleveland: IELF and EBM showed moderate calibration, while XGBoost displayed overconfidence at mid-range probabilities. IELF offered better balance, aligning closer to the diagonal reference.
- Framingham: All models showed poorer calibration than Cleveland. IELF outperformed XGBoost. EBM remained most stable at the cost of underestimating positive risk.
- These results demonstrate that IELF achieves calibration close to clinically acceptable standards while preserving predictive strength (Figure 18).

### 3.8 Stability of feature rankings

To quantify robustness, we computed Kendall’s τ and Overlap@10 across CV folds.

- Cleveland: Rankings were moderately stable, with τ ≈ 0.74–0.79 and overlap ≈0.79–0.88.
- Framingham: Rankings were more consistent, particularly for IELF (τ = 0.838, overlap = 0.92). This highlights IELF’s resilience against instability when applied to larger, more heterogeneous datasets.
  IELF’s enhanced generalization stems from three complementary design choices:
- nested cross-validation strictly separates tuning and evaluation, preventing information leakage.
- ensemble fusion dynamically balances bias and variance through data-driven weighting of EBM and XGBoost; and
- stability-validated explanations (Kendall’s τ and Overlap@ k) ensure consistent feature attribution across folds, mitigating overfitting to dataset-specific noise.

### 3.9 Generalization and stability

The observed consistency of feature rankings and explanation attributions across folds indicates that IELF generalizes more effectively than single or non-interpretable ensemble models. By combining EBM’s additive transparency with XGBoost’s gradient-boosted precision, IELF captures both stable global patterns and fine-grained nonlinearities without overfitting to specific folds. Nested cross-validation further constrains hyperparameter tuning to the inner loop, preventing information leakage and ensuring that outer-fold scores reflect true out-of-sample performance. This design yields lower variance across repetitions, stronger calibration, and balanced recall–precision trade-offs in independent cohorts, demonstrating that IELF’s predictive behavior is robust, reproducible, and generalizable across heterogeneous populations.

### 3.10 Subgroup analyses

We further evaluated fairness and clinical consistency by stratifying performance by sex, age bins, smoking status, and diabetes status.

- Cleveland: IELF achieved strong AUC values (>0.94) across both sexes and perfect calibration in younger age groups. Older patients exhibited reduced calibration, but IELF preserved balanced recall and precision. As expected, some Cleveland subgroups (e.g., sex = 0, younger age bins) showed near-perfect AUC values. These reflect the small subgroup sizes in this dataset, which inflate variance and limit generalizability. Results should therefore be interpreted with caution, while the larger Framingham subgroups provide a more reliable view of fairness and subgroup performance.
- Framingham: Performance was more heterogeneous. IELF maintained competitive AUC values in subgroups such as diabetes = 0 and nonsmokers, but recall dropped sharply in smoker and diabetic subgroups. These findings emphasize the need for subgroup-sensitive validation in clinical deployment.

Taken together, these results show IELF’s stability in Cleveland and highlight the need for subgroup-sensitive validation in heterogeneous cohorts like Framingham.

### 3.11 State-of-the-art comparison

Comparison of IELF with state-of-the-art approaches for heart disease prediction.

A quantitative comparison of IELF with recent state-of-the-art approaches for heart disease prediction is summarized in Table 12. Performance metrics are reported as published (best per study) and may not be directly comparable due to differences in preprocessing, data splitting, and validation strategies. In particular, several studies report results after resampling or feature engineering before dataset splitting, which can introduce data leakage and inflate performance estimates. IELF results are from held-out test sets with thresholds tuned via nested CV. Interpretability indicates whether the model provides intrinsic transparency or relies on post-hoc explanations.

**TABLE 12:** Comparison of IELF with recent state-of-the-art (SOTA) approaches for heart disease prediction. Reported metrics are as published (best per study) and are not directly comparable due to differences in preprocessing, resampling, and validation strategies. In several high-accuracy reports (e.g., Talukder et al., 2025; Moreno-Sánchez, 2020; Alamatsaz et al., 2024), resampling/feature processing is described but fold-restriction is not always explicit; when such steps occur before the train/test split or outside CV folds, this can introduce information leakage and inflate accuracy. By contrast, Mienye and Jere (2024) explicitly use a split-first design with 5-fold CV confined to the training set, so we do not classify their workflow as leakage-prone. IELF (this work) was evaluated with 5 × 5 nested CV, train-only resampling (SMOTE confined to training folds), and included probability calibration and subgroup checks, prioritizing reproducibility and interpretability over maximal point accuracy (see Results & Discussion for analysis of our lower headline accuracy).

Study/Year	Dataset(s)	Best algorithm	Preprocessing/Features	Accuracy (%)	Precision (%)	Recall (%)	F1-score (%)	AUC (%)	Interpretability
Mienye and Jere. (2024)	Cleveland, framingham	XGBoost	SHAP; Bayesian optimization; 5-fold CV	97.1/92.1	98.9 (Clev)	97.1	–	–	Post-hoc (SHAP)
Moreno-Sanchez. (2020)	UCI heart failure	XGBoost	Feature selection (RFE, MI, ANOVA); SHAP; GridSearchCV	83.0	–	–	–	–	Post-hoc (SHAP)
Alamatsaz et al. (2024)	MIT-BIH & LTAF arrhythmia	CNN + LSTM hybrid	Noise filtering, normalization, resampling; SHAP	98.2	–	∼98	–	–	Post-hoc (SHAP)
Talukder et al. (2025)	Cleveland, framingham	HxAI-ML (hybrid XAI)	Combined resampling + hybrid feature extraction; multi-optimizer	98.7/98.7	98.8	99.2	98.7	99.7	Hybrid explainability
Ghose et al. (2024)	UCI heart, framingham	Stacked EBM + XGBoost + SHAP	Bayesian optimization; statistical validation	95.2/91.8	94.6	92.7	93.6	94.1	Hybrid (SHAP + intrinsic)
IELF (this work), 2025	Cleveland	EBM + XGBoost ensemble	Scaling; encoding; imputation; nested CV tuning	88.5	83.9	92.9	88.1	96.0	Intrinsic (EBM) + post-hoc (SHAP)
IELF (this work), 2025	Framingham	EBM + XGBoost ensemble	SMOTE; Scaling; encoding; imputation; nested CV tuning	82.6	34.4	16.3	22.1	68.6	Intrinsic (EBM) + post-hoc (SHAP)

Compared to Ghose et al. (2024), IELF reports slightly lower headline accuracies but introduces local LIME explanations, formal reproducibility documentation, and statistical rank-based validation absent in their study, thereby extending methodological rigor rather than predictive extremity. Thus, whereas Ghose et al. (2024) established the viability of stacked explainable ensembles for heart-disease prediction, the proposed IELF framework constitutes the first statistically validated interpretable ensemble that quantifies explanation stability under nested cross-validation. Recent works on cardiovascular disease prediction report remarkably high accuracies, often exceeding 97%–99% on benchmark datasets. For instance, Mienye and Jere (2024) attained 97.1% on Cleveland and 92.1% on Framingham under XGBoost with Bayesian optimization and post-hoc explanation with SHAP. For clarity, we do not classify Mienye and Jere (2024) as at risk of leakage; their Methods indicate a split-first design with 5-fold CV performed only within the training set during Bayesian optimization. Their method highlights the merits of hyperparameter-optimized ensembles for achieving optimal predictive precision, yet does not test for calibration, subgroup fairness, nor explanation reproducibility over folds. In comparison, IELF incorporates an inherently interpretable model (EBM) with XGBoost under a soft-voting ensemble, measures explanation stability (Kendall’s τ, Overlap@10), and includes calibration and fairness analysis under nested cross-validation. Methodologically, this approach results in less aggressive accuracy rates (88.5% Cleveland; 82.6% Framingham) yet guarantees explanation reproducibility and clinical verifiability at the expense of showcasing just another state-of-the-art ensemble. Most recently, Talukder, Talaat and Kazi (2025) introduced HxAI-ML, a hybrid explainable model combining deep learning and machine learning pipelines. Their approach reported near-perfect accuracies of 98.7%–99.5% with AUC ≈99.7, substantially higher than conventional ensembles and boosting methods.

While these results are impressive, they raise important methodological considerations in some reports. Some ≥97–99% reports are based on vigorous resampling/feature-processing pipelines; if such steps are carried out pre-split or not evidently confined to training folds, then data leakage will inflate accuracy. Talukder et al. (2025) illustrate several balancing variants (RO, SMOTE, Tomek links, IHT) but do not indicate fold-restricted resampling. Mienye and Jere (2024) split first then use 5-fold CV within training during Bayesian optimization. As such, direct comparisons must account for when the preprocessing/resampling takes place as well as whether nested/fold-scoped model selection is implemented.

Mienye and Jere (2024) highlight in particular the capability of optimized ensembles, such as XGBoost, Random Forest, and AdaBoost with Bayesian tuning, complemented by SHAP-based interpretation. Nevertheless, their inquiry, like many others, is mostly focused on predictive accuracy improvement at the expense of calibration examination, subgroup fairness, and even the explainability’s reproducibility.

Conversely, the IELF framework was specifically constructed to highlight clinical reliability and reproducibility. Our approach insisted upon:

- Strict separation of training and test data, avoiding pre-split resampling or feature engineering that can inflate accuracy (a risk in some prior studies such as Talukder et al., 2025; Moreno-Sánchez, 2020; Alamatsaz et al., 2024).
- Nested cross-validation for hyperparameter tuning, providing unbiased generalization estimates rather than single-split optimization.
- Calibration and fairness analyses, which were not addressed in Mienye & Jere’s study but are essential for deployment in heterogeneous clinical populations; and
- Quantitative validation of explanation stability (Kendall’s τ, Overlap@10), extending beyond post-hoc SHAP analysis to ensure consistent and reproducible interpretability.

By quantifying explanation stability and incorporating dual local interpretability (SHAP + LIME), IELF reframes the contribution as the first statistically validated interpretable ensemble with quantitative explanation-stability and reproducibility metrics. Under these stricter methodological controls, IELF achieved 96.0% AUC on Cleveland and 68.6% AUC on Framingham, alongside interpretable feature rankings and subgroup analyses. While headline accuracies were lower than those reported for HxAI-ML, IELF uniquely delivered a reproducible, interpretable, and fair ensemble framework. This positions IELF not simply as a competitor chasing maximum accuracy, but as a trustworthy benchmark for developing clinically viable AI where transparency and reliability are as critical as raw predictive power.

## 4 Conclusion

This study introduced the Interpretable Ensemble Learning Framework (IELF), combining Explainable Boosting Machines (EBM) and XGBoost with SHAP-based explanations to enhance cardiovascular disease prediction. Across the Cleveland and Framingham datasets, IELF consistently provided competitive performance, achieving 96.0% AUC on Cleveland and 68.6% AUC on Framingham, while simultaneously delivering transparent and clinically grounded explanations. By integrating intrinsic interpretability (EBM) with post-hoc analysis (SHAP), IELF addresses one of the central barriers to clinical adoption of AI models: trustworthiness and explainability.

Compared to more recent state-of-the-art techniques like Talukder et al.'s HxAI-ML (2025) reporting over 99% accuracies and 99.7% AUC, IELF reveals a distinct and supplementary contribution. Though HxAI-ML obtains higher headline results, its use of pre-split resampling and multi-optimizer pipelines poses risks of overestimated performance. Likewise, Mienye and Jere (2024) optimized Random Forest, AdaBoost, and XGBoost via Bayesian hyperparameter tuning and interpreted models via SHAP attaining near-perfect sensitivity and specificity for Cleveland and Framingham datasets. Their work betrays the promise of accuracy-optimizing ensembles but does not tackle calibration, subgroup fairness, nor explanation stability. IELF, conversely, is first (as far as we know) to unite EBM and XGBoost with SHAP and offer quantative stability verification of explanations (Kendall’s τ, Overlap@10) under strict nested cross-validation, in addition to calibration and subgroup fairness verification. These choices inevitably reduce maximum performance but ensure reproducibility, fairness, and clinical reliability, dimensions often underreported in models optimized solely for accuracy. IELF thus complements high-performing but less transparent approaches, positioning itself as a trustworthy benchmark for translational AI in healthcare.

The findings suggest that IELF could function as a clinically viable decision-support tool, particularly in contexts where transparency is as critical as accuracy. Explanations grounded in established cardiovascular risk factors (e.g., age, blood pressure, cholesterol, diabetes, smoking) allow outputs to be directly mapped onto clinical guidelines, enhancing clinician trust. With integration into electronic health records and deployment in screening programs, IELF has the potential to support earlier risk detection, personalized interventions, and improved patient outcomes.

## 5 Limitations

This study has several limitations. Both datasets (Cleveland and Framingham) are dated and not fully representative of contemporary, diverse populations. The relatively small size of Cleveland introduces variance in subgroup analyses, while harmonization between datasets required simplifications that may obscure population-specific patterns. Finally, although SHAP and EBM provided consistent explanations, their interpretability remains dataset-dependent and may vary in other clinical contexts. SMOTE oversampling was applied only to the Framingham dataset to address imbalance; the Cleveland dataset was not oversampled due to its natural class balance.

## 6 Future work

Future extensions will focus on validating IELF in larger, multi-center, and demographically diverse cohorts to assess fairness and generalizability. Incorporating temporal data (e.g., longitudinal follow-up) and multimodal information (e.g., imaging, genomics, electronic health records) represents a natural methodological advance. Crucially, prospective clinical studies are needed to evaluate IELF’s real-world impact on decision-making, workflow integration, and patient outcomes. Continued benchmarking against high-performing but less transparent models such as HxAI-ML will clarify the trade-offs between maximal accuracy and trustworthy interpretability in practice.

## Data Availability

Publicly available datasets were analyzed in this study. This data can be found here: https://www.kaggle.com/datasets/noeyislearning/framingham-heart-study and https://www.kaggle.com/datasets/ritwikb3/heart-disease-cleveland.
